# Identification of Components of the Hippo Pathway in *Hydra* and Potential Role of YAP in Cell Division and Differentiation

**DOI:** 10.3389/fgene.2021.676182

**Published:** 2021-10-06

**Authors:** Manu Unni, Puli Chandramouli Reddy, Mrinmoy Pal, Irit Sagi, Sanjeev Galande

**Affiliations:** ^1^ Centre of Excellence in Epigenetics, Department of Biology, Indian Institute of Science Education and Research, Pune, India; ^2^ Department of Biological Regulation, Weizmann Institute of Science, Rehovot, Israel; ^3^ Department of Life Sciences, Shiv Nadar University, Delhi-NCR, India

**Keywords:** hydra, hippo pathway, YAP, budding, cell prolferation

## Abstract

The Hippo signaling pathway has been shown to be involved in regulating cellular identity, cell/tissue size maintenance and mechanotransduction. The Hippo pathway consists of a kinase cascade which determines the nucleo-cytoplasmic localization of YAP in the cell. YAP is the effector protein in the Hippo pathway, which acts as a transcriptional cofactor for TEAD. Phosphorylation of YAP upon activation of the Hippo pathway prevents it from entering the nucleus and abrogates its function in the transcription of the target genes. In Cnidaria, the information on the regulatory roles of the Hippo pathway is virtually lacking. Here, we report the existence of a complete set of Hippo pathway core components in Hydra for the first time. By studying their phylogeny and domain organization, we report evolutionary conservation of the components of the Hippo pathway. Protein modelling suggested the conservation of YAP-TEAD interaction in *Hydra*. Further, we characterized the expression pattern of the homologs of *yap, hippo, mob* and *sav* in *Hydra* using whole-mount RNA *in situ* hybridization and report their possible role in stem cell maintenance. Immunofluorescence assay revealed that *Hvul*_YAP expressing cells occur in clusters in the body column and are excluded in the terminally differentiated regions. Actively proliferating cells marked by Ki67 exhibit YAP colocalization in their nuclei. Strikingly, a subset of these colocalized cells is actively recruited to the newly developing bud. Disruption of the YAP-TEAD interaction increased the budding rate indicating a critical role of YAP in regulating cell proliferation in *Hydra.* Collectively, we posit that the Hippo pathway is an essential signaling system in *Hydra*; its components are ubiquitously expressed in the *Hydra* body column and play a crucial role in *Hydra* tissue homeostasis.

## Introduction

Multicellularity arose about 400–1000 million years ago on earth (Butterfield, 2000) independently in at least 16 different eukaryotic lineages, leading to complex multicellular taxa like metazoa, Fungi and Embryophyta (Butterfield, 2000; Brunet and King, 2017). Considering the bilaterians as the most complex and diverse multicellular clade, a basic “Developmental Toolkit” required for the generation, organization and maintenance of multicellular structures can be assessed. The origin of these developmental tools, including transcription factors, signaling pathways, cell adhesion, and cell polarity-related genes, can be traced back to basal metazoans (Tweedt and Erwin, 2015). A detailed analysis of these development toolkits, body plan and differential germ layers and a diverse cell-type system indicates Cnidarians are arguably the first phylum to evolve and exhibit features that underlie the traits commonly seen in Bilateria. Cnidarians exhibit an oral-aboral body axis polarity with a diploblastic germ layer organization. These germ layers in cnidarians have been reported to form myoepithelial cells, nerve-net of sensory/ganglion neuronal cells, gastric cells, germline cells and cnidocytes, which are the defining feature of the phylum. Studies in the past few decades have clearly shown that these primitive organisms display highly complex developmental programs and toolkits commonly found in the bilaterians.

Among the cnidarians, *Hydra* is the best-characterized model. *Hydra* is a freshwater polyp known to exhibit tremendous regenerating potential with a capability to regenerate even from reaggregated cells of dissociated polyps (Gierer et al., 1972). It has been a classical model for developmental and regeneration biology for more than two centuries and has contributed immensely towards the understanding of morphogen mediated processes and understanding various cell signaling pathways (Reddy P. C. et al., 2019). Among the 10 developmentally important signaling pathways- Notch, Wnt, Hedgehog, TGFβ/BMP, Receptor-tyrosine kinase (RTK), Hippo, NF-κB, JAK-STAT, JNK & Nuclear receptor signaling pathway family, seven of them have been shown to be functional according to the studies on *Hydra*. Many components of the Wnt signaling have been reported in *Hydra* and their role has been established to be important in the regulation of head organizer activity (Hobmayer et al., 2000). Many components of Notch signaling are present in *Hydra* and have been reported to be important in the boundary formation in tissues (Sprinzak et al., 2010; Münder et al., 2013). The TGFβ superfamily of signaling pathways has also been reported to be crucial for *Hydra* developmental signaling such as during tentacle formation, foot formation, symmetry breaking (Reinhardt et al., 2004; Rentzsch et al., 2007; Watanabe et al., 2014). Members of the RTK family of signaling pathways - VEGF, FGF and Ephrin have been shown to be crucial for regeneration in *Hydra* (Tischer et al., 2013; Krishnapati and Ghaskadbi, 2014). NF-κB has been reported to be essential for early regenerative time points in *Hydra* (Franzenburg et al., 2012; Wenger et al., 2014). While their role is presently thought to be innate immunity/inflammation-related, its direct developmental regulation is yet to be established. JNK in *Hydra* is crucial in nematocyte differentiation and regulation of TLR-signaling (Philipp et al., 2005; Franzenburg et al., 2012). Among the nuclear receptor family of signaling pathways, Retinoblastoma gene has been found to be expressed in almost all cell types in *Hydra* but its specific role has not been deciphered (Schenkelaars et al., 2018). Another nuclear receptor protein, NR3E has been found to be expressed in *Hydra* and is predicted to respond to the parasterol A, a cnidarian A-ring aromatic steroid (Khalturin et al., 2018). Among the three remaining developmentally important signaling pathways yet to be reported in *Hydra* are- Hedgehog, JAK-STAT and Hippo signaling.

The hippo pathway has emerged as a major player in orchestrating spatio-temporal regulation of cell differentiation, proliferation, tissue size control, and apoptosis. These capabilities enable the Hippo pathway to be important in regulating morphogenesis and tissue or organ regeneration. It was first described and reported in *Drosophila* while screening for tumor suppressor genes in 1995 (Xu et al., 1995). However, it was only in 2005 when Yorkie (Yki), a transcription co-activator, was linked to Hippo signaling, the importance of the Hippo pathway in regulating transcriptional landscape was truly realized (Huang et al., 2005). Yes-associated protein, also known as YAP, is a highly conserved mammalian homolog of the *Drosophila* Yki. The Hippo core components are kinases that phosphorylate YAP through a cascade, which represses its transcriptional activity by preventing its nuclear transportation and hence its interaction with transcription factors like TEAD (Fulford et al., 2018). Upon phosphorylation, YAP is sequestered in the cytoplasm through 14-3-3 interaction or undergo ubiquitination for its degradation. The core components of the Hippo pathway characterized in *Drosophila* consists of Ser/Thr kinases- Hippo (Hpo) and Warts (Wts); and their adapter proteins- Salvador (Sav) and Mats. The equivalent set of factors in mammalians is named as- Mst, Lats, Sav and Mob, respectively. The Hippo pathway and its functions are highly conserved between invertebrates and vertebrates at the cellular and molecular levels.

There has been a paucity of literature to date about Hippo signaling in basal metazoans. The role of Hippo signaling in highly regenerative organisms like *Hydra* is unknown. A recent study in another cnidarian reported that *Clytia hemispherica* has all the core components of the Hippo pathway, and CheYki has cell proliferation regulatory function. Hence, it is pertinent to characterize the homologs of core Hippo pathway components in *Hydra* to understand their role in *Hydra* regeneration and cell proliferation and differentiation. A recent study has reported the presence of core components of the Hippo pathway in *Nematostella* (Hilman and Gat, 2011), suggesting that this pathway has relatively conserved ancient origin during the evolution of multicellular organisms. Here, using a combination of bioinformatic analysis and molecular cloning, we report the existence of a complete set of core Hippo pathway components in *Hydra.* Using domain analysis and 3D protein modelling, we show that these homologs have a conserved domain and motif architecture, indicating a possible conserved interactive signaling network. Whole-mount *in situ* hybridization (WISH) analysis revealed that these genes are expressed with a few gene-specific variations across the body column. Adapting CheYki specific antibody for the immunofluorescence assay of Hydra YAP, we show that a subset of nuclear localized YAP occurs in clusters of cells across the body column with no expression in the terminally differentiated regions. We also show that most of the actively proliferating cells in *Hydra* have nuclear-localized YAP expressing cells, and a subset of these are recruited to developing buds. Further, we report the existence of a separate non-clustered non-proliferating nuclear-localized YAP expressing cell population at the hypostomal region, which may be involved in oral fate specification and maintenance. Finally, we show that disruption of YAP-TEAD interaction with verteporfin leads to increased budding rate in polyps indicating an ancient and conserved role of YAP in tissue homeostasis.

## Materials and Methods

### Animal Culture

Clonal culture of *Hydra vulgaris* Ind-Pune (Reddy et al., 2011) was maintained in *Hydra* medium by following standard methods at 18 ± 1°C (Horibata et al., 2004). Polyps were fed daily with freshly hatched Artemia nauplii larvae and washed 6–8 h after feeding. For the regeneration experiment, *Hydra* polyps starved for 24 h were decapitated just below the tentacle base and allowed to regenerate till 0, 1, 2, 4 and 8 h post-amputation (hpa). The polyps were then fixed and processed for immunofluorescence assay. For the budding experiment, *Hydra* polyps starved for 24 h were collected at different stages of bud development. These were then fixed and processed for immunofluorescence assay. The different stages of budding were identified and labelled as reported previously (Otto and Campbell, 1977).

### Identification of Hippo Pathway Homologs in *Hydra*

*Hydra magnipapillata* genome draft comprising 82.5% of 1.05 Gbp sequenced genome available as Refseq was initially used for identifying Hippo Pathway core components (Chapman et al., 2010). This assembly turned out to be incomplete, and we were unable to fish out any homologs. Therefore, an in-house transcriptome assembly generated in the Galande laboratory (Reddy PC. et al., 2019) was used for the present study. The in-house transcriptome was merged with the NCBI RefSeq to improve the assembly further to generate a hybrid assembly. The hybrid assembly was found to be 99.6% complete as compared to 95.7% exhibited by NCBI RefSeq (Reddy PC. et al., 2019). Using the stand-alone NCBI BLAST program, hits of homologs of Hippo pathway core components were identified (Madden, 2013). To confirm the hits, Reverse BLAST was performed. Finding a hit of a homolog in different phyla or species would confirm the homolog status. To further confirm, the amino acid sequences of these homologs were searched in HMMER (Hmmer, RRID:SCR_005305) for affirmation based on the hits returned (Potter et al., 2018). Once the homologs were identified, they were further analyzed for domain organization by SMART (SMART, RRID:SCR_005026) (Letunic et al., 2002). After manual evaluation of the domain organization, the domain architecture was constructed to scale using DOG 2.0 software (Ren et al., 2009).

### Molecular Phylogenetic Trees

Sequences from different representative phyla were collected based on protein BLAST searches using the Human YAP sequence as a query. The collected sequences were aligned using MUSCLE (Edgar, 2004). The alignment was trimmed using an automated trimAl programme (Capella-Gutiérrez et al., 2009). This alignment was subjected to phylogenetic analysis using FastTree 2 to generate an approximately maximum likelihood (ML) tree (Price et al., 2010). This method was selected after testing PhyML and RaxML as FastTree 2 has given better confidence on branching points and this could be due to the highly divergent nature of the sequences. This pipeline was implemented in the online platform NGphylogeny.fr (Lemoine et al., 2018). Here, the LG substitution model was used with Felsenstein’s phylogenetic bootstrap with 1000 (Lemoine et al., 2019). The phylogenetic tree was visualized by using the iTOL web server (Letunic and Bork, 2019). The tree was rooted using *Amphimedon queenslandica* YAP-like sequence as an outgroup. The domain organization analysis and visualization were carried out using DoMosaics software (Moore et al., 2014) using embedded HMMER3 tools (Mistry et al., 2013) and Pfam data. The sequences details were provided in Supplementary Table S1.

To analyse the rest of the Hippo pathway components, alignments and molecular phylogenetic trees of the protein sequences were carried out using MEGA 6.0 software (Tamura et al., 2013). MUSCLE algorithm was used for amino acid sequence alignment (Edgar, 2004). The alignment was graphically represented using Jalview (Waterhouse et al., 2009).

### Cloning of Hippo Pathway Homologs From *Hydra*

Total RNA was extracted from *Hydra* polyps starved for 48 h and cDNA was synthesized from total RNA using Improm-II reverse transcriptase system (Promega™) according to the manufacturer’s instructions. Hippo pathway genes were amplified by polymerase chain reaction using Pfu DNA polymerase with the following primers:

*Hvul_yap*_forward:5′-ATGGATATGAATTCTACGCAACGGC-3′,
reverse: 5′-CTA​CAA​CCA​AGT​CAT​ATA​TGC​ATT​AGG​C-3′;
*Hvul_tead*_forward: 5′-ATG​GCG​GAA​AAC​TGT​CGA​GAT​CC-3′,
reverse: 5′-TCA​GTC​TCT​GAC​TAA​TTT​AAA​TAT​GTG​GT-3′;
*Hvul_hpo*_forward: 5′-ATG​TCT​CGC​AGT​TTG​AAG​AAG​TTG​AG-3′,
reverse: 5′-TTA​AAA​ATT​TGC​TTG​CCT​GCG​TT-3′;
*Hvul_mob*_forward: 5′-ATG​AGT​TTC​CTG​TTT​GGC​TCC​A-3′,
reverse: 5′-TTA​TTT​ATT​AAT​TAA​CTT​ATC​CAT​AAG​TTC-3′;
*Hvul_lats*_ forward: 5′-ATG​GCA​GCT​AAT​AAT​CTT​TTT​AGT​AG-3′,
reverse: 5′-TCA​TAC​AAA​AAC​AGG​CAA​CTT​GC-3′;
*Hvul_sav*_forward: 5′-ATG​TTT​AAG​AAA​AAA​GAT​ATT​ATC​AAA​ACA-3′,
reverse: 5′-TTA​AAC​ATG​AGT​TTT​TTT​AAA​AGA​AAT​ACT-3′

The PCR conditions: Initial denaturation at 94°C for 5 min, followed by 30 cycles of denaturation at 94°C for 30 s, annealing at the respective annealing temperatures (Ta) for 45 s and extension at 72°C for 45 s with the final extension 72°C for 5 min. The PCR amplified products were gel eluted using Mini elute kit (Qiagen), followed by A-tailing reaction using KapaTaq enzyme and cloned in pGemT-Easy vector system (Promega™) or TOPO TA cloning vector as per the manufacturer’s instructions. The recombinant plasmids were sequenced using sequencing primers, and the nucleotide sequences of cloned genes were deposited at NCBI Genbank (*Hvul_yap*- MW650883; *Hvul_tead*- MW650884; *Hvul_hpo*- MW650879; *Hvul_mob*- MW650880; *Hvul_lats*- MW650881 and *Hvul_sav*- MW650882).

### Whole-mount *In Situ* Hybridization

Digoxigenin-labelled sense and antisense RNA probes were prepared by *in vitro* transcriptions using recombinant plasmids of target genes made as mentioned above (Roche Life Science) and used for *in situ* hybridization. Whole-mount *in situ* hybridization was performed on the polyps as described by Martinez et al. (1997) with the following changes (Martinez et al., 1997). The animals were relaxed for 2 min in 2% urethane. Treatment with proteinase-K was performed for an optimum of 15 min, and heat-inactivation of the endogenous alkaline phosphatases was done at 70°C for 15 min in 1X SSC. Digoxigenin labelled RNA probes at a 200–600 ng/ml concentration of the probe was used for hybridization at 59°C. The post-hybridization washes were performed using 1X SSC-HS gradients. After staining with BM-purple AP substrate for 30 min–1 h at room temperature, the animals were mounted in 80% glycerol for imaging. Imaging was carried out using a ×10 DIC objective lens with Axio Imager Z1 (Zeiss).

### Cryosectioning of WISH Stained *Hydra* Samples

The stained polyps were rehydrated to PBS gradually through PBS: methanol gradient (25, 50, 75, and 100% wash each for 10 min). These polyps were then shifted to a 30% sucrose solution by gradually taking it through 10 and 20% for 30 min each. The polyps were left in 30% sucrose overnight. These polyps were then embedded in 10% PVP (polyvinyl pyrrolidone) by making cubes of PVP (1 × 1 × 2 cm^3^) made from aluminium foil cast. The embedded polyps were then sectioned (25 µm thick) using Leica CM1950 – Cryostat. The sectioned ribbons were collected on a glass slide and covered and sealed under a coverslip. The sections were then photographed under ZEISS Axio Zoom V16 apotome microscope.

### Analysis of Expression of Hippo Pathway Components From Single-Cell Transcriptome Profile

t-SNE plots and gene expression plots of Hippo pathway components were generated and extracted from the Single Cell Portal (https://portals.broadinstitute.org/single_cell/study/SCP260/stem-cell-differentiation-trajectories-in-Hydra-resolved-at-single-cell-resolution). In order to use the Single Cell Portal, gene IDs of Hippo pathway components were acquired through a BLAST search in the Juliano aepLRv2 nucleotide database *via Hydra* 2.0 Genome Project Portal (https://research.nhgri.nih.gov/Hydra/sequenceserver/). To determine the clusters of cells that express individual Hippo pathway components, differential gene expression was analyzed using edgeR, a tool to analyze RNA-seq data using the trimmed mean of M-values (TMM) method. Differential gene expression was calculated as fold change.

### *Hydra* Cell Dissociation

Cells from *Hydra* polyps were dissociated using a protocol described previously with minor modifications (Greber et al., 1992). The Pronase enzyme was replaced by more efficient, pure and negligible endotoxin-containing Liberase™ TL (Roche/Sigma Aldrich). The enzyme was used at a concentration of 0.2 U/ml. Dissociation was performed either in *Hydra* dissociation medium (Greber et al., 1992) with a slight modification of replacing TES with HEPES (15 mM) for buffering. Fifty *Hydra* polyps were washed twice in a sterile-filtered *Hydra* medium and transferred into a 1.5 ml tube. The medium was removed, and 1 ml Liberase in dissociation medium was added. Cells were dissociated for 90 min at room temperature (22–24°C) with gentle agitation on a nutator and followed by gentle pipetting.

### Immunofluorescence Staining

A recently published paper reported the presence of Yorkie (YAP/Yki) in *Clytia hemispherica* and producing polyclonal antibody specific to CheYki in rabbit against the peptide FNRRTTWDDPRKAHS (Coste et al., 2016). This antibody along with the pre-immune serum was kindly gifted by Dr Michaël Manuel (Sorbonne Universités, Université Pierre et Marie Curie (UPMC), Institut de Biologie Paris-Seine (IBPS) CNRS). The antibody was validated by immunofluorescence analysis (the antibody doesn’t work for western blotting experiments). Proliferating cells in *Hydra* were identified using anti-Ki67 antibody [OTI5D7] (ab156956; Abcam).

Immunofluorescence assay was performed as per the protocol is given in Takaku et al., 2014 (Takaku et al., 2014). *Hydra* polyps were starved at least for 1 day before fixation. Animals were relaxed in 2% urethane for 1–2 min and fixed in 4% paraformaldehyde (in 1XPBS) overnight at 4°C or 1 h at RT. 1:100 concentration of primary antibody was used. 1:100 concentration of Invitrogen Alexa-conjugated secondary antibodies were used. Invitrogen Alexa 488 conjugated Phalloidin for staining. DAPI was used for nuclear staining. These samples were then imaged on ZEISS Axio Zoom V16 (for regeneration and whole animal images) or Andor Dragonfly Spinning Disc (for budding *Hydra*) microscopes.

### Fluorescent Flow Cytometry Using ImageStream

The dissociated cells from *Hydra* were fixed and stained as described above, using an anti-CheYki antibody. DNA was counterstained using DAPI. Cells were imaged using multispectral imaging flow cytometry (ImageStreamX flow-cytometer; Amnis Corp., Seattle, WA, United States). For multispectral imaging flow cytometry, ∼1 × 10^5^ cells were collected from each sample and data were analyzed using image analysis software (IDEAS 4.0; Amnis Corp.). Images were compensated for fluorescent dye overlap by using single-stained controls. Cells were gated for single cells, using the “area” and “aspect ratio” features, and for focused cells, using the Gradient RMS feature, as previously described (George et al., 2006). Cells were gated for cell size- Large and Small based on the area feature. Cells with an area less than 200 μm^2^ was labelled as “Small” and anything bigger as “Large.” The large cells were found to be non-significant when compared to the cells found in the negative control (due to autofluorescence). The “Small” cells were further gated for nuclear vs cytoplasmic localization. The “similarity” feature was used to achieve this by colocalizing the YAP (Yki) signal with the DAPI signal. Cells with a similarity value greater than two were considered nuclear-localized, and cells with a low similarity value of less than two were considered cytoplasmically localized (*See* Supplementary Figure S8).

### Verteporfin Treatment and Budding Assay

Verteporfin (Vp) was procured from Sigma (SML0534) and dissolved in DMSO. A 2 mM stock was prepared and kept in 50 µl aliquots at −20 C for long-term storage in the dark. The vial was thawed for 30 min in the dark before use. For the assay, the polyps were treated with 5 μM Vp in *Hydra* medium and the same volume of DMSO in *Hydra* medium as vehicle control. For the budding assay, the non-budding polyps were collected and incubated in Vp. The Vp solution was replaced every 24 h, and readings for number buds were taken every 24 h. Every step of the Vp treatment assay was performed in the dark. For statistical analysis, the average number of buds per polyp was calculated by measuring the total number of buds from a set of 48 polyps and then divided by 48. For this experiment, three such biological replicates were done (*N* = 3) with 48 polyps per set (*n* = 48). Hence a total of 144 polyps were used for the experiment per treatment. For detachment assay, the total number of buds fallen till the specified day was calculated by adding the number of buds fallen on each previous day. This experiment was done using 48 polyps. Statistical significance of the data was calculated by measuring *p*-value using Heteroscedastic two-tailed *t*-test (Supplementary Table S2 budding kinetics tab for statistical data).

### Modelling

The 2.8 A° crystal structure of the human YAP-TEAD complex deposited on PDB (4RE1) was as a reference for modelling the TEAD binding domain and YAP binding domains of *Hydra* YAP-TEAD complex (Zhou et al., 2015). Modeller software (Webb and Sali, 2016) was used to build five optimum models based on 4RE1 in the multi-model mode. Among the five models, the model with minimum DOPE assessment score and maximum GA341 assessment score was chosen for the final analysis. The model was then visualized in CHIMERA for analysis, superpositioning and annotation (Pettersen et al., 2004). The non-covalent bond analysis was performed in Biovia Discovery Studio Visualizer (BIOVIA, 2017). The Binding energy calculations done using PRODIGY web server (Xue et al., 2016).

## Results

### Characterization and Phylogenetic Analysis of *Hydra* Hippo Pathway Genes

**FIGURE 1:**
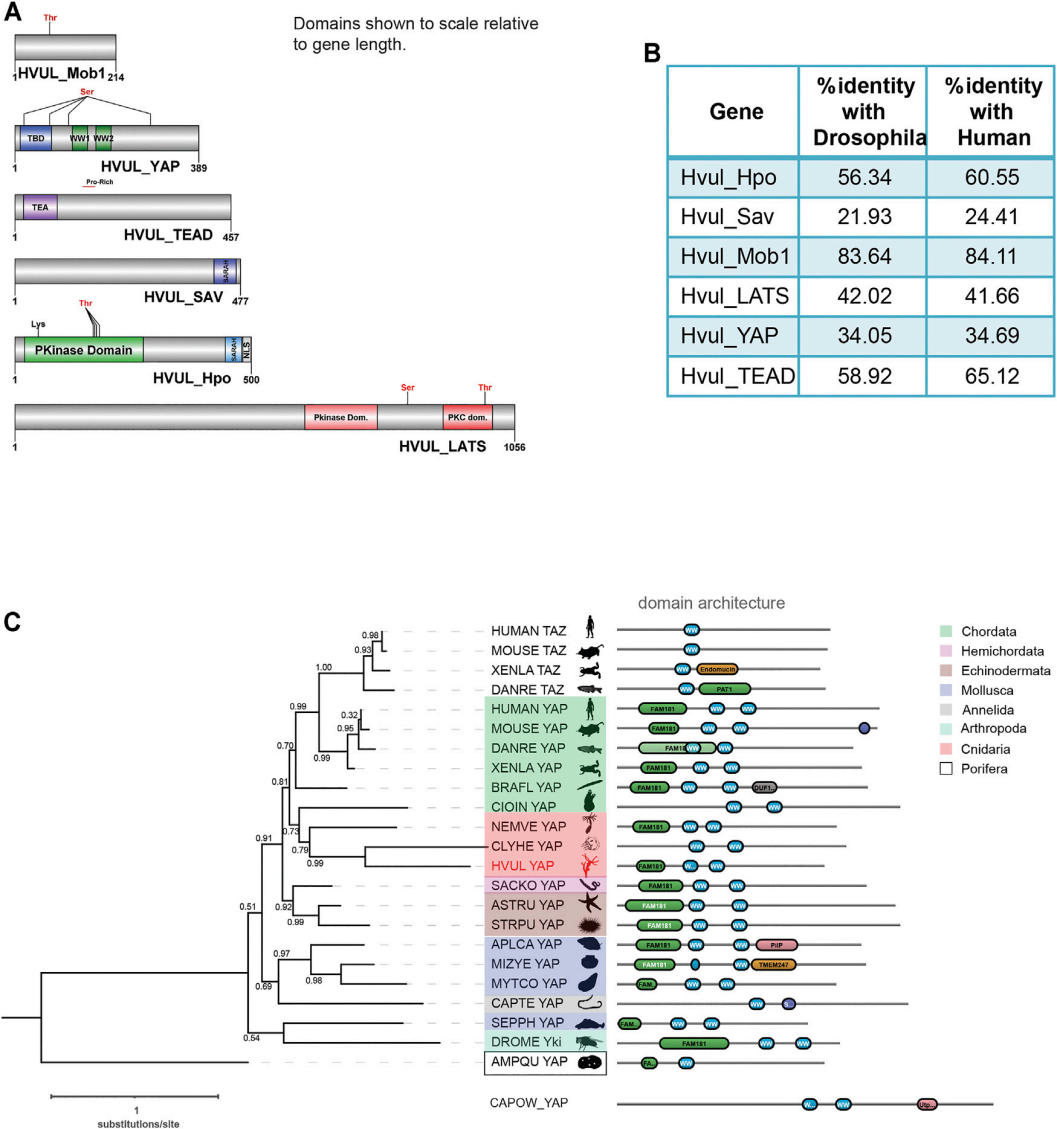
Identification of Hippo pathway homologs in *Hydra*. **(A)** Domain architecture of the homologs as visualized using DOG 2.0. **(B)** Depicts the percent identity of *Hydra* homolog with *Drosophila* and Human. **(C)** Phylogenetic tree and domain organization of YAP homologs across the animal phyla. The phylogenetic analysis was carried out on NGphylogeny.fr webserver and the tree was generated using FastTree 2 method. Here, the phylogenetic tree was rooted at Amphimedon queenslandica YAP-like sequence (AMPQU). Domain organization analysis was carried out using DoMosaics software. Branch support values are displayed at the branching points. Different phyla are highlighted with distinct colours. *Hydra* YAP homologue (*HVUL* YAP) is highlighted in red colour font. A UNIPROT style abbreviations for organism names are used. Sequence details are provided in the Supplementary Table S4.

Core Hippo pathway homologs–*hippo/mst, mob, lats, sav, yap and tead* were identified from the in-house *Hydra* transcriptome using NCBI stand-alone BLAST (Reddy PC. et al., 2019). In mammals, *hippo, mob, lats* and *yap* have two paralogs each while *tead* has four paralogs. *Sav*, on the other hand, has no reported paralogs. The occurrence of these paralogs has been attributed to whole-genome duplication events correlated to certain fish species (Chen et al., 2019). Therefore, any species that evolved earlier than fishes do not contain paralogs reported for Hippo pathway genes. Conforming to these reports, *Hydra* consists of only one gene coding for each core Hippo pathway component. The *Hydra* Hippo pathway homologs were labelled as *Hvul_hpo, Hvul_mob, Hvul_lats, Hvul_sav, Hvul_yap* and *Hvul_tead*. These homologs in *Hydra* were confirmed by obtaining the corresponding amplicons from *Hydra* cDNA (Supplementary Figure S1). Upon determining the nucleotide percent identity with other reported model organisms used for studying the Hippo pathway, we find that *Hydra* had a higher percent identity with humans than *Drosophila* (Figure 1B). *Hvul_hpo* shows about 60% identity with humans and 56% identity with *Drosophila*. *Hvul_sav* is comparatively less conserved with a 24% identity with human and 22% identity with *Drosophila*. *Hvul_mob* is highly conserved across the animal phyla with about 84% identity with humans and 83% identity with *Drosophila*. *Hvul_lats* shares 41.6% identity with humans and 42% identity with *Drosophila*. *Hvul_yap* shows 34.6% identity with humans and 34% identity with *Drosophila*. *Hvul_tead* exhibits about 65% identity with humans and 59% identity with *Drosophila*.

An earlier study performed the phylogenetic analysis of YAP homologues found in selective phyla (Hilman and Gat, 2011). However, this analysis did not cover the majority of invertebrate phyla such as Annelida, Mollusca and Echinodermata. This could be due to a lack of reliable data for the identification of the YAP homologues. Here, we have combined the phylogenetic analysis with predicted domain architecture. We used a YAP-like sequence found in *Amphimedon queenslandica* as an outgroup for rooting the tree. Additionally, a protein sequence with BLAST similarity from a unicellular Eukaryote (C*apsaspora owczarzaki*) was used for domain organization comparison. In this analysis, we observed that the *Hydra* homologue of YAP exhibits a strong affinity to the chordate counterparts rather than non-chordate homologues (Figure 1C). An interesting observation after the inclusion of multiple invertebrate phyla in the analysis is that they are highly diverged compared to the Cnidarian and Chordata species. This can be interpreted based on the invalid branch support values (Figure 1C). Additionally, a molluscan homologue, *Sepia pharaonic* (SEPPH_YAP), showed more similarity with *Drosophila* Yki and S*accoglossus kowalevskii* homologue (SACKO_YAP) showed more similarity with Echinodermata homologues (Figure 1C). Domain organization analysis has led to the identification of variability in the N-terminal homology domain (FAM181). This region contains TEAD binding domain (TBD). Surprisingly, in *Capitella teleta* (Annelida), *Clytia hemisphaerica* (Cnidaria), and *Ciona intestinalis* (Chordata), the FAM181 domain could not be detected (Figure 1C). This could be due to the higher sequence divergence in this region.

A detailed domain analysis using SMART website for the amino acid sequence of Hippo pathway homologs revealed a highly conserved domain organization of the proteins analyzed, indicating a fully functional pathway consisting of these core components (Figure 1A). *Hvul*_HPO domain analysis revealed a conserved N-terminal Protein kinase domain (PKinase Domain) and a C-terminal SARAH (Salvador-RASSF-Hippo) domain. The presence of these domains indicates the conserved regulation of activation of *Hvul*_HPO kinase activity (Glantschnig et al., 2002; Praskova et al., 2004; Boggiano et al., 2011). The *Hvul*_SAV also can be seen to have conserved the SARAH domain required for orchestrating the reported scaffolding activity (Yin et al., 2013). *Hvul*_LATS domain architecture indicates conservation of the hydrophobic motif (Motif: AFYEFTFRHFFDDGG) (a 40% hydrophobicity confirmed using web-based peptide analysis tool at www.peptide2.com/N_peptide_hydrophobicity_hydrophilicity.php) containing the Threonine residue (T993) required for the activation of LATS by HIPPO phosphorylation (T1079 in humans) (Supplementary Figure S2A) (Hergovich et al., 2006; Ni et al., 2015). The MOB binding motif is highly conserved in *Hvul*_LATS as compared to the human and mouse (Supplementary Figure S2B). The auto-activation T-loop (near S909 in humans) of *Hvul*_LATS is 100% conserved (Motif: AHSLVGTPNYIAPEVL) near S830 (Supplementary Figure S2B) (Ni et al., 2015). *Hvul*_MOB is highly conserved (84% identity with human MOB) as compared to any other components of Hippo pathway homologs in *Hydra*, indicating highly conserved function. The same site as reported for Human MOB is also highly conserved in *Hvul*_MOB at T35 (Motif: LLKHAEATLGSGNLR) (Supplementary Figure S2C). This site is crucial for the release of the LATS-MOB complex from the MST-SAV-LATS-MOB complex and further initiation of LATS auto-activation (Ni et al., 2015). *Hvul*_YAP domain analysis revealed that it had a conserved TEAD-Binding Domain (TBD) and two WW domains. A serine phosphorylation prediction for YAP primary sequence was performed using GPS 2.1 web-based tool (Xue et al., 2010). Based on GPS prediction and manual curation, *Hvul*_YAP is predicted to have an LATS phosphorylation site at S74 (motif: PIHTRAR**S**LPSNIGQ) and S276 (motif: YTAYMN**S**SVLGRGSS) homologous to the S127 (motif: PQHVRAH**S**SPASLQL) and S381 (motif: SDPFLN**S**GTYHSRDES) (Supplementary Figure S2D). Similar to mammals, a phosphodegron motif (DSGLDG) was identified immediately downstream to the S276 site (S381 in humans), which could be phosphorylated by CK1-ɣ at S287 (S388 in humans) of *Hvul*_YAP (Supplementary Figure S2E) (Zhao et al., 2010). These analyses indicated that the Hippo pathway effector protein YAP is well equipped for regulation by the LATS and CK1-ɣ. With its defined TEAD binding domain and WW domain, it could interact with transcription factor TEAD and other reported PPXY domain-containing proteins.

### Structural Features of YAP and TEAD Interaction

The Hippo effector protein YAP is known to elicit its biological function as a transcription co-effector by interacting with transcription factors. Presently, YAP is known to interact with TEAD, β-catenin, SMAD, RUNX, p73 and ErbB4 for regulating their transcriptional responses as an activator or repressor (Strano et al., 2001; Komuro et al., 2003; Zhao et al., 2008; Szeto et al., 2016; Passaniti et al., 2017; Pan et al., 2018). Among these, YAP-TEAD interaction has been extensively studied and is known to be important for regulating cell growth and size and tissue architecture (Totaro et al., 2018). The interaction of YAP and TEAD was first shown to form through their specific interaction domains in 2001 (Vassilev et al., 2001). The structural features of this interaction in humans were first demonstrated in 2009, showing how the TEAD binding domain (TBD) in YAP (amino acids 53–99) interacted with the YAP binding domain (YBD) in the TEAD (position: amino acids 209–426) (Li et al., 2010). The YBD consists of 12 β strands that arrange into two β sheets in an opposing fashion to form a β-sandwich fold. The four α helices from the YBD are arranged at the two ends of the β-sandwich fold for stabilizing the structure. The study showed that TBD-YBD interaction occurs over three interfaces. Each interface consisted of one of the following secondary structure of the TBD-the β1 strand, α1 helix or α 2 helix responsible for interacting with the globular YBD of the TEAD at the C-terminal. It was shown that the β1 strand of TBD interacted with the β7 strand of the YBD (interface 1), The α1 helix from TBD interacted with α3 and α4 helices of the YBD (interface 2). The α2 of the TBD was bound to the YBD through its interaction with α1 and α2 helices (interface 3) (Li et al., 2010).

**FIGURE 2:**
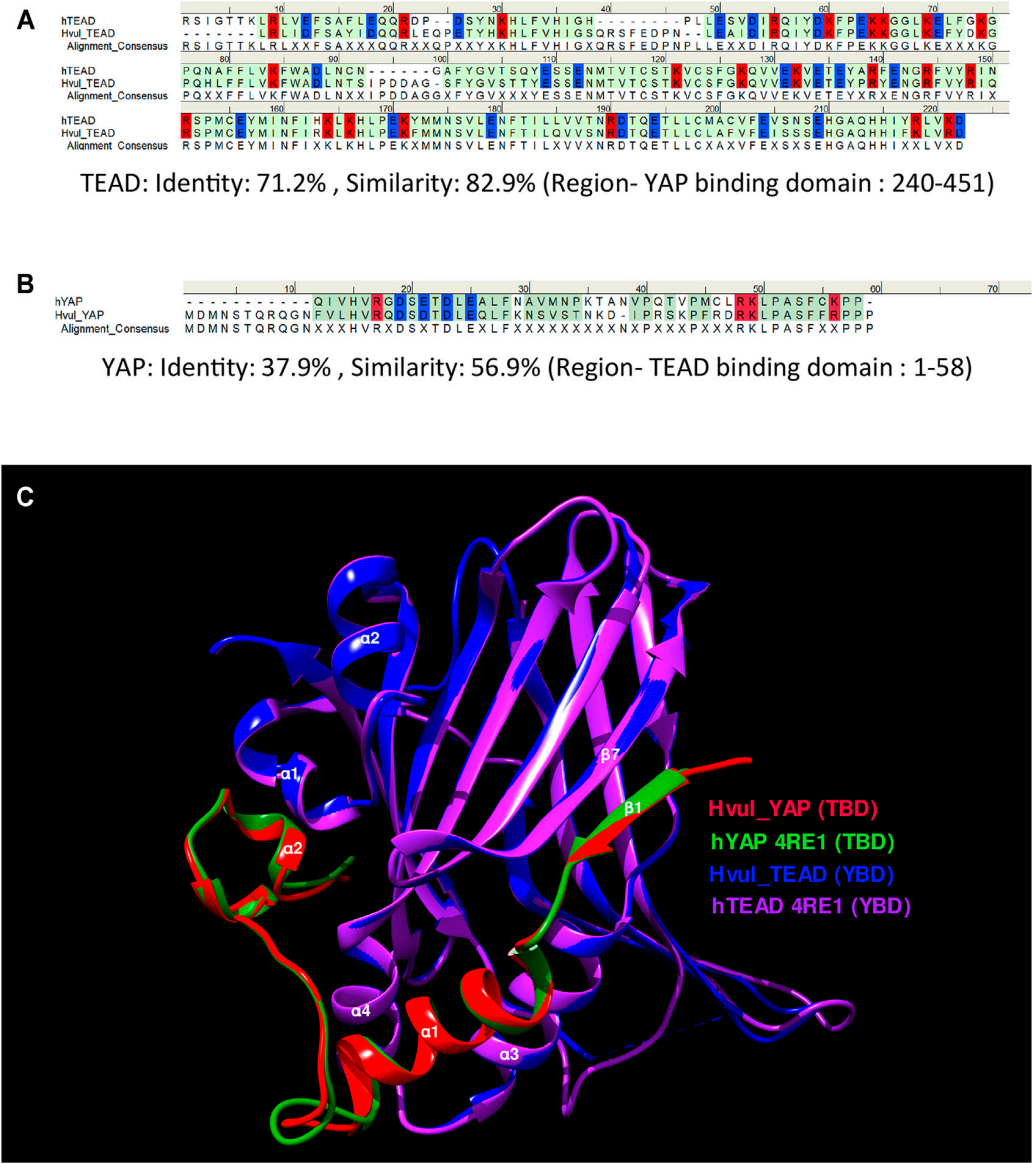
YAP-TEAD interaction domain is structurally conserved in *Hydra*. **(A)** Sequence alignment of human and *Hydra* TEAD YAP-binding domains (YBD) showing 71.2% sequence identity. **(B)** Sequence alignment of human and *Hydra* YAP TEAD-binding domains (TBD) showing 37.9% sequence identity. The alignment consensus shows conserved amino acid residues at a given position. If the there is no conservation, the position is labelled as X. Colour code: amino acid residues with positive charge-red, negative charge-blue and neutral-green. **(C)** Structural superposition of predicted *Hvul*_TEAD YBD and *Hvul*_YAP TBD with YBD and TBD complex in Human (PDB:4RE1) showing highly conserved β-strands and α-helices structural placement. Important α-helices and β-strands are indicated with their number identification which are involved in the interaction of YBD and TBD. Colour code: Red- *Hvul*_YAP TBD, Blue- *HVUL*_TEAD YBD, Green-human YAP TBD (PDB ID-4RE1), Purple-human TEAD YBD (PDB ID-4RE1). D) Interaction of YBD (green color) with the TBD (purple color) in *Hydra* modelled using 4RE1 structure showing how the globular YBD (depicted in surface features) is bound by TBD (depicted as ribbon) through interactions at three different regions -region 1, region 2, and region 3. The amino acid side chains from TBD are represented as sticks for understanding their role in the interaction.

Amino acid sequence alignment of the predicted YBD (amino acids 240–251) and predicted TBD (position: 1–58) of *Hvul*_YAP and *Hvul*_TEAD respectively with Human YAP and TEAD revealed 71.2% sequence identity (82.9% sequence similarity) of YBD (Figure 2A) and a 37.9% sequence identity (56.9% sequence similarity) of TBD (Figure 2B) which indicates plausible structural conservation and hence interacting capability of TBD with YBD. To confirm the same, the 3D structure of the YBD and TBD of *Hvul*_YAP and *Hvul*_TEAD was modelled using MODELLER software (Webb and Sali, 2016). The modelling was done based on the 4RE1 X-ray diffraction structure deposited at Research Collaboratory for Structural Bioinformatics PDB (RCSB PDB-https://www.rcsb.org/) models the interaction of human homologs of TBD and YBD at a resolution of 2.20 Å. The model generated from the *Hydra* homologs was superimposed on the human YAP (hYAP) and hTEAD structure from 4RE1 and was found to be highly structurally similar (RMSD for *Hvul*_YAP:hYAP- 0.338 A° and for *Hvul*_TEAD:hTEAD- 0.310 A°) and indicated a conserved interaction capability of *Hvul*_YAP and *Hvul*_TEAD (Figure 2C). The modelled YBD-TBD complex of *Hydra* clearly shows how three different regions- Region 1, Region 2 and Region three of TBD (purple) interacts with the globular YBD (green) by non-covalent bond interactions (Supplementary Figure S3A). The Region 1 interface consisting of TBD β1 (amino acids 10–17) and YBD β7 (358–363) strands interact with seven hydrogen bonds in the human complex, forming an anti-parallel β sheet (Li et al., 2010). In *Hydra* counterpart, there are only six hydrogen bonds (green dotted lines) due to the presence of Gln18 in β1 instead of Gly59 found in humans (Li et al., 2010), introducing a rotation in the preceding Arg, which disables it from forming a hydrogen bond (Supplementary Figure S3B). The 2^nd^ interface (Region 2) has the α1 helix of the TBD (amino acids 20–32) fitting right into the binding groove of the YBD formed by the α3 and α4 helices of the YBD (amino acids 385–409) (Supplementary Figure S3C). Similar to humans, this region is mainly mediated by hydrophobic interactions with the α1 helix of the TBD having conserved LXXLF motif for hydrophobic groove binding (Li et al., 2010). This interaction mainly consists of Leu24, Leu27and Phe28 from TBD and Try386, Lys393 and Val406 of YBD (pink dotted lines). In *Hydra*, few hydrogen bonds (green dotted lines) not found in humans may lead to a more stable interface. The third region (third interface) consists of a twisted coil and α2 helix (amino acids 42–58) from the TBD interacting deeply with the pocket formed by the α1 helix, β4, β11 and β12 helices of the YBD. This region was found to be indispensable for the YAP-TEAD complex formation in humans (Li et al., 2010). Region three in *Hydra* contains the hydrophobic side chains of the TBD–Phe44 (Met86 in humans), Leu49, Pro50 and Phe53, forming extensive van der Waals interactions with the YBD of TEAD at Glu280, Ala281, Ile282, Gln286, Ile287, Leu312, Leu316, Val431, His444 and Phe446 (Supplementary Figure S3D). The interface is further strengthened by multiple hydrogen bonds (indicated in green dotted lines) – TBD_Arg47:YBD_Gln286, TBD_Lys48:YBD_Gln286, TBD_Ser52:YBD_Glu280 and YBD_Lys314:TBD_Phe53. The hydrophobic interactions in region three consist of Phe53, Pro50 and Phe44 from TBD and Lys314, Glu408 and Phe446 from the YBD. In comparison, the hydrophobic interactions involving Pro56 and Pro57 from TBD with Trp316 and His444 respectively help to push the proline residues out of the hydrophobic pocket. One of the unique aspects that can be predicted from the model is that the *Hydra* region three YAP-TEAD complex can form two salt bridges (orange dotted lines) - TBD_Arg47:YBD_Asp289:YBD_Asp289 and TBD_Lys48:YBD_Asp283:YBD_Asp451. The human complex only forms a salt bridge at TBD_Arg89:YBD_Asp249: YBD_Asp249. These observations indicate a more stable YAP-TEAD interaction in *Hydra* as compared to humans. To shed more light on the same, the computationally calculated binding energy of the YAP-TEAD complex between the two organisms were compared using the web-based server PRODIGY (PROtein binDIng enerGY prediction) in Protein-protein mode (Xue et al., 2016). The ΔG of the YAP-TEAD complex in humans is about −6.8 kcal mol^−1,^ while the complex in *Hydra* has a value of −14.7 kcal mol^−1^. This large difference in the binding energy supports the possibility that the YAP-TEAD complex in *Hydra* is much more stable.

### Expression Analysis of the Hippo Pathway Genes in *Hydra*

#### *Hvul_yap* Expression in *Hydra*

**FIGURE 3:**
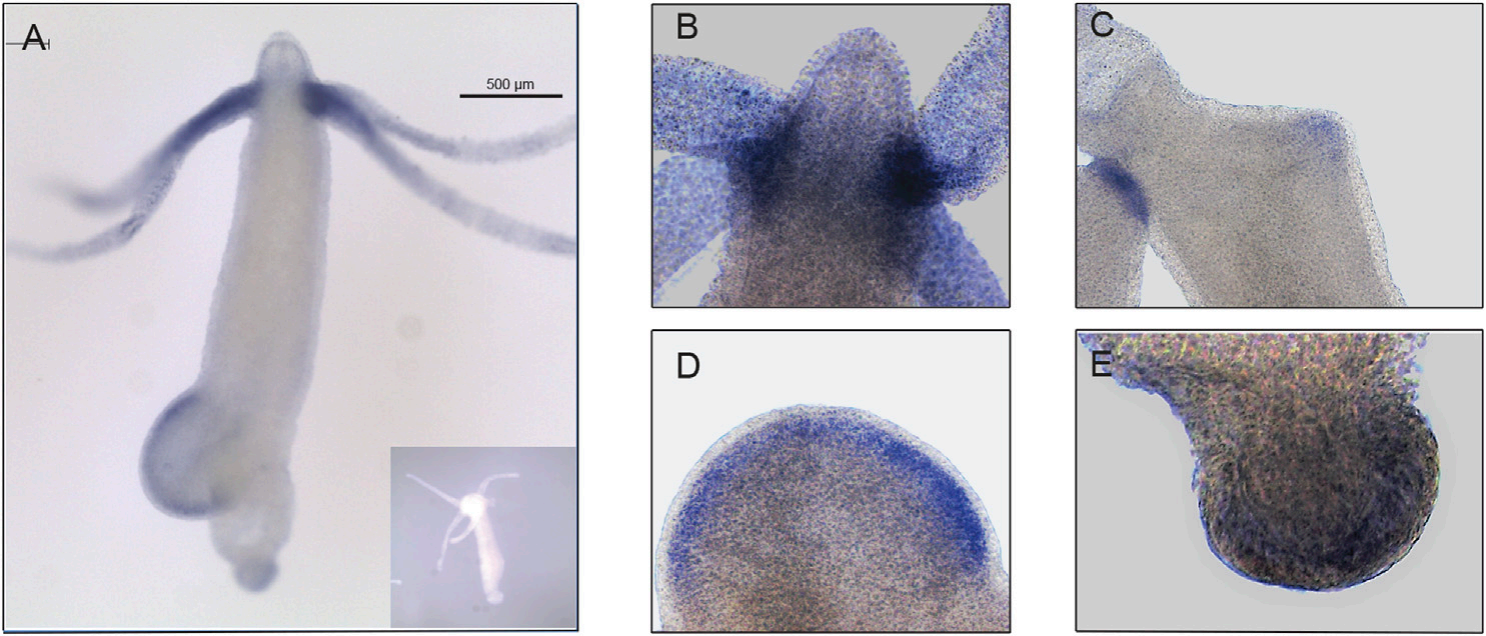
*Hvul_yap* expression analysis in *Hydra*. Whole-mount *in situ* hybridization of *Hvul_yap* expression at **(A)** Region across the polyp (inset shows polyp probed with a sense RNA probe). **(B)** Head, **(C)** early bud/late bud foot, **(D).** mid-stage bud, **(E)** basal disk (The scale bar is 500 µm long, *N* = 3).

The expression pattern of *Hvul_yap* in *Hydra* polyp was studied by whole-mount *in situ* hybridization (WISH). The staining pattern observed from the whole polyp indicates low-level expression throughout the body with higher expression at the tentacle base and tip of the early stages of the developing new bud (Figure 3A). A closer look indicates that the expression is more robust in the endodermal cells than in the ectodermal cells (Figures 3B–E). *yap* expression in the early stages of bud development indicates its role in budding. Higher *yap* expression at the region of high mechanical stress such as the tentacle base, early budding tip and mature bud-parent polyp boundary indicates a probable ancient mechano-sensory role of YAP in *Hydra*. These polyps were cryosectioned to obtain a closer look at the types of cells expressing *yap* (Supplementary Figure S4). The images of these sections revealed cells in doublets, quadruplets and groups of cells, among other stained cells indicating their interstitial stem cell origin, plausibly nematoblast and nests of nematoblasts.

### Expression Pattern of *Hvul_hpo, Hvul_mob* and *Hvul_sav* Genes

**FIGURE 4:**
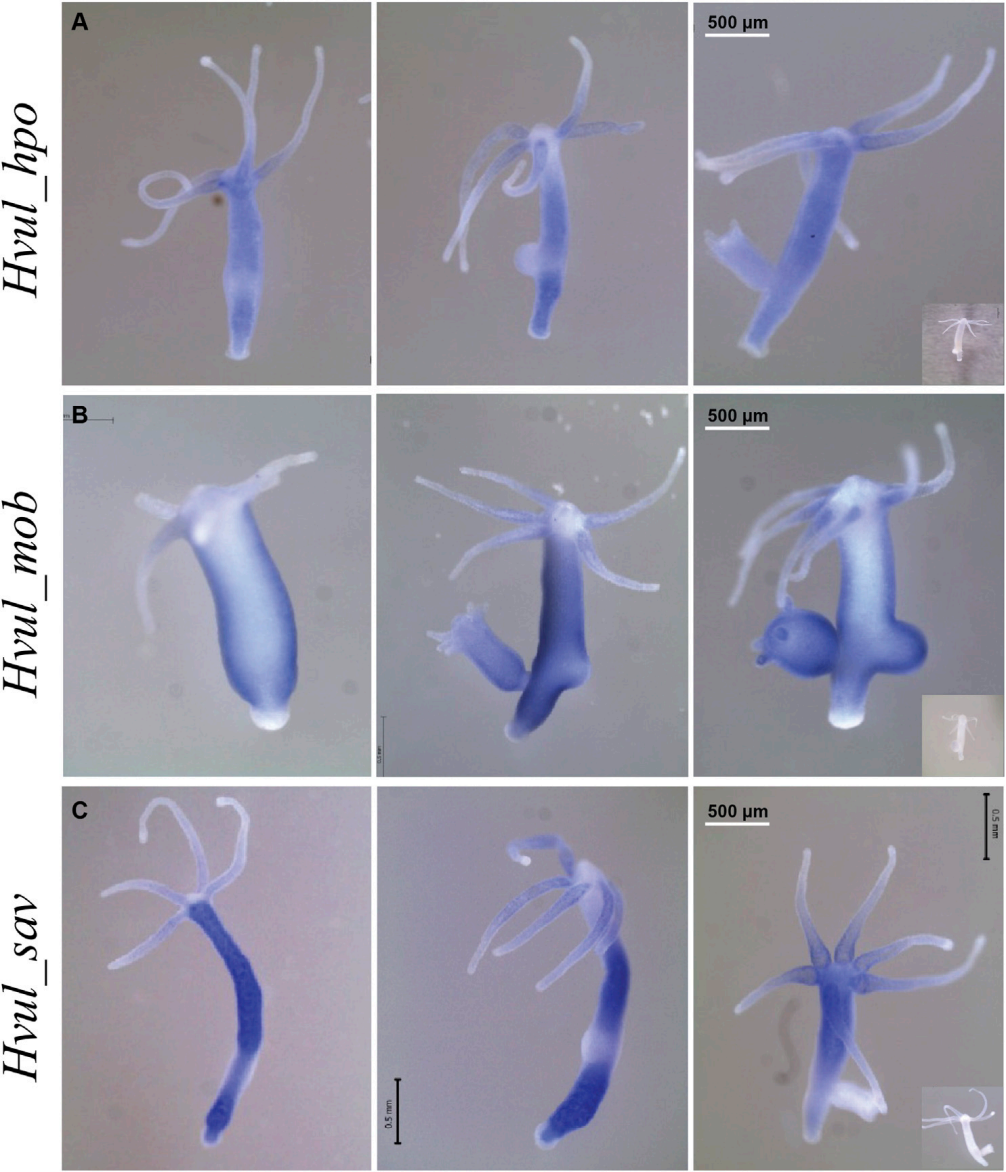
*Hvul_hpo*, *Hvul_mob* and *Hvul_sav* expression analysis in *Hydra*. Whole mount *in situ* hybridization of **(A)** *Hvul_hpo*, **(B)** *Hvul_mob* and **(C)** *Hvul_sav* expression for the whole polyp. The insets on the right indicate negative controls probed with sense RNA probe. (The scale bar is 500 µm long, *N* = 3).

An RNA WISH study of *Hvul_hpo* showed expression throughout the gastric region (Figure 4A). No expression was observed at the differentiated zones of hypostome, tentacle or basal disk, which might indicate a role in stem-cell maintenance or differentiation but not in terminally differentiated cells. There is a slight reduction in expression at the budding zone and early buds, which might indicate the antagonistic role of HPO towards YAP activity in areas of high mechanical stress as reported in other organisms. *Hvul_hpo* expression can also be seen at mature bud-parent polyp boundary, indicating a fine-tuning of regulation of Hippo pathway-dependent during bud detachment. *Hvul_mob* expression showed a similar pattern to *Hvul_yap* with a distinct down-regulation at the basal disk region of both adult and budding *Hydra* (Figure 4B). *Hvul_sav* expression reflected the expression pattern of *Hvul_hpo,* indicating a similar role. It can also be noted that there is a marked reduction in expression at the budding region, early and late buds, unlike the *Hvul_hpo* (Figure 4C).

A recent study reported high-throughput sequencing of the transcriptome of 24,985 single *Hydra* cells using Drop-seq and identified the molecular signatures of various cell states and types (Siebert et al., 2019). The differential expression of the Hippo pathway components and their pattern were examined using the Single Cell Portal. The expression patterns of *Hvul_yap, Hvul_tead, Hvul_hpo, Hvul_lats, Hvul_sav & Hvul_mob* were queried. From the single-cell data, *Hvul_yap* expression was insignificantly dysregulated or differentially expressed between cell types (Supplementary Figure S5). Surprisingly, such a trend was commonly observed between all the other Hippo pathway components, namely, *Hvul_tead, Hvul_hpo, Hvul_lats, Hvul_sav & Hvul_mob*. This indicates a slight disparity with the WISH data. This could be due to the lack of enough resolution from the datasets used. The data shown here only represent a relative fold-change between the cells and may indicate that the expression levels are relatively the same. The WISH data also indicate that most of the cells express almost all types of Hippo pathway components, yet at the same time, we see that they are excluded from some regions. These may be highly stage-specific and hence difficult to be picked up in sc-RNAseq of whole polyps. Nevertheless, the findings from analyzing single-cell data argue in favor of the fact that the Hippo pathway components are essential for cells and need to be expressed in almost all cell types. Their activity might be regulated at the protein level, and hence a protein-based analysis is essential to better understand the regulation of Hippo pathway in *Hydra*.

### Protein Expression Analysis of the *Hvul*_YAP in *Hydra*

#### Region-specific and Cell-type Expression of *Hvul*_YAP in *Hydra*

The *Clytia hemispherica* specific Yorkie (CheYki) antibody was raised against a peptide from the WW1 region of CheYorkie in rabbit (Coste et al., 2016). The CheYki peptide sequence was extracted from the Marine Invertebrate Model Database (MARIMBA) and was used to align with *Hvul*_YAP using CLUSTAL Omega. The full protein alignment showed just a 39.36% identity. However, a peptide-specific (immunogen) alignment gave a 60% identity which raised the probability of cross-reactivity of this antibody against *Hvul*_YAP (Supplementary Figure S6A). A BLAST search of the CheYki immunogen peptide sequence returned only two hits with an E-value less than 0.01. Both of the hits were *Hydra* YAP (Supplementary Table S3). An immunofluorescence assay (IFA) was run using CheYki antibody or pre-immune serum to test the same. The IFA yielded a robust signal for the CheYki antibody compared to the negative control (Supplementary Figure S6B). Examination of localization of YAP expressing cells revealed a pattern similar to what we found in YAP ISH (Supplementary Figure S5). The expression was seen more or less throughout the body. The tentacle base showed high expression similar to that seen in ISH, but the number of YAP expressing cells drops in the hypostomal region and the inter-tentacle zone. Unlike the pattern of transcripts seen in the ISH, the YAP expressing cells were depleted at the basal disk region.

**FIGURE 5:**
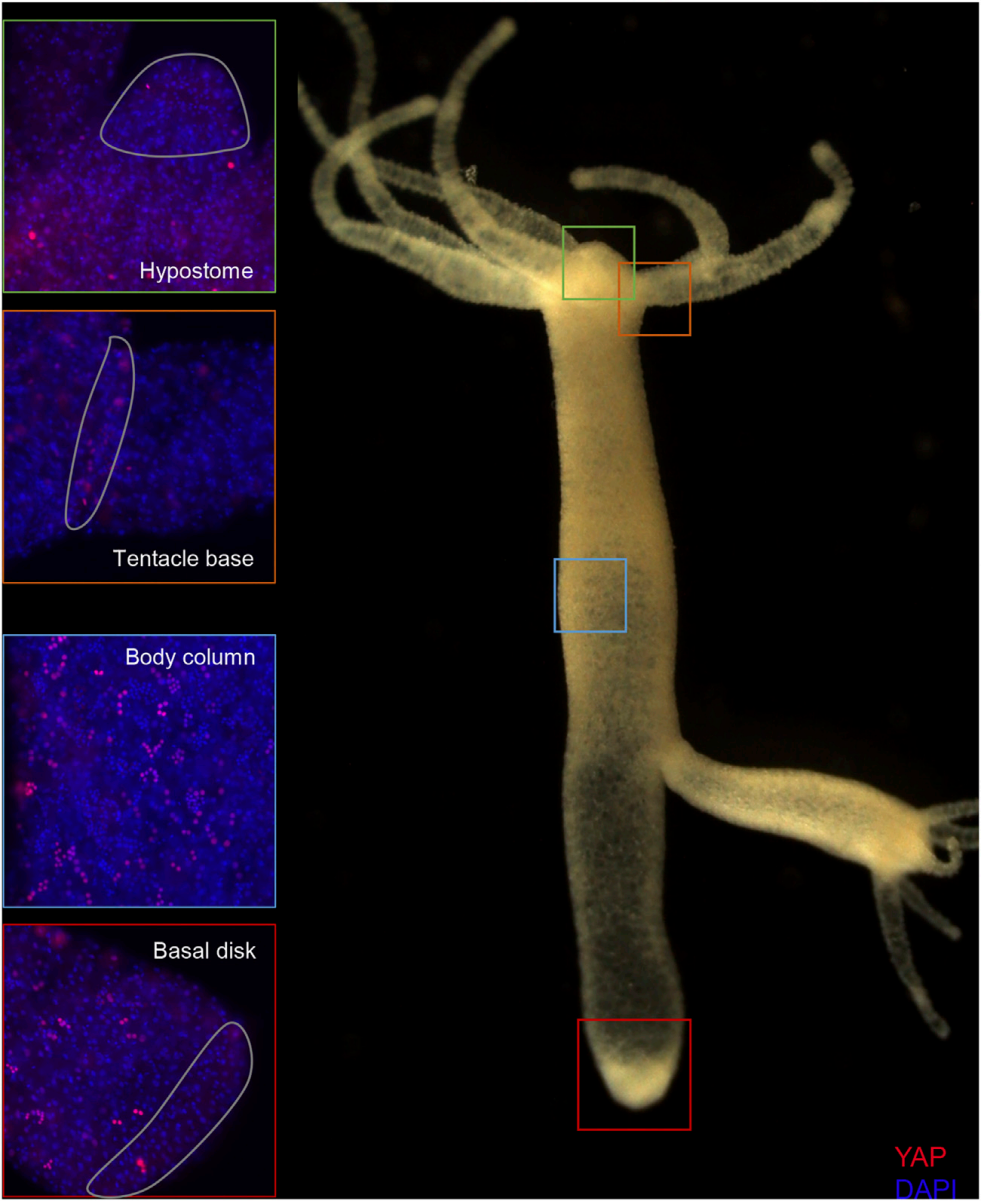
Expression of *Hvul*_YAP in *Hydra*. Immunofluorescence assay of *Hvul*_YAP performed using anti-CheYki antibody showing localization of YAP positive cells at various locations in an adult polyp. The red fluorescent dye shows Alexa 594 staining of YAP and the blue dye shows DAPI staining of nucleus. The hypostomal region is indicated by a green box, the tentacle base is indicated by an orange box. The body column is indicated by a blue box and basal disc area is indicated by a red box. (*N* = 3).

**FIGURE 6:**
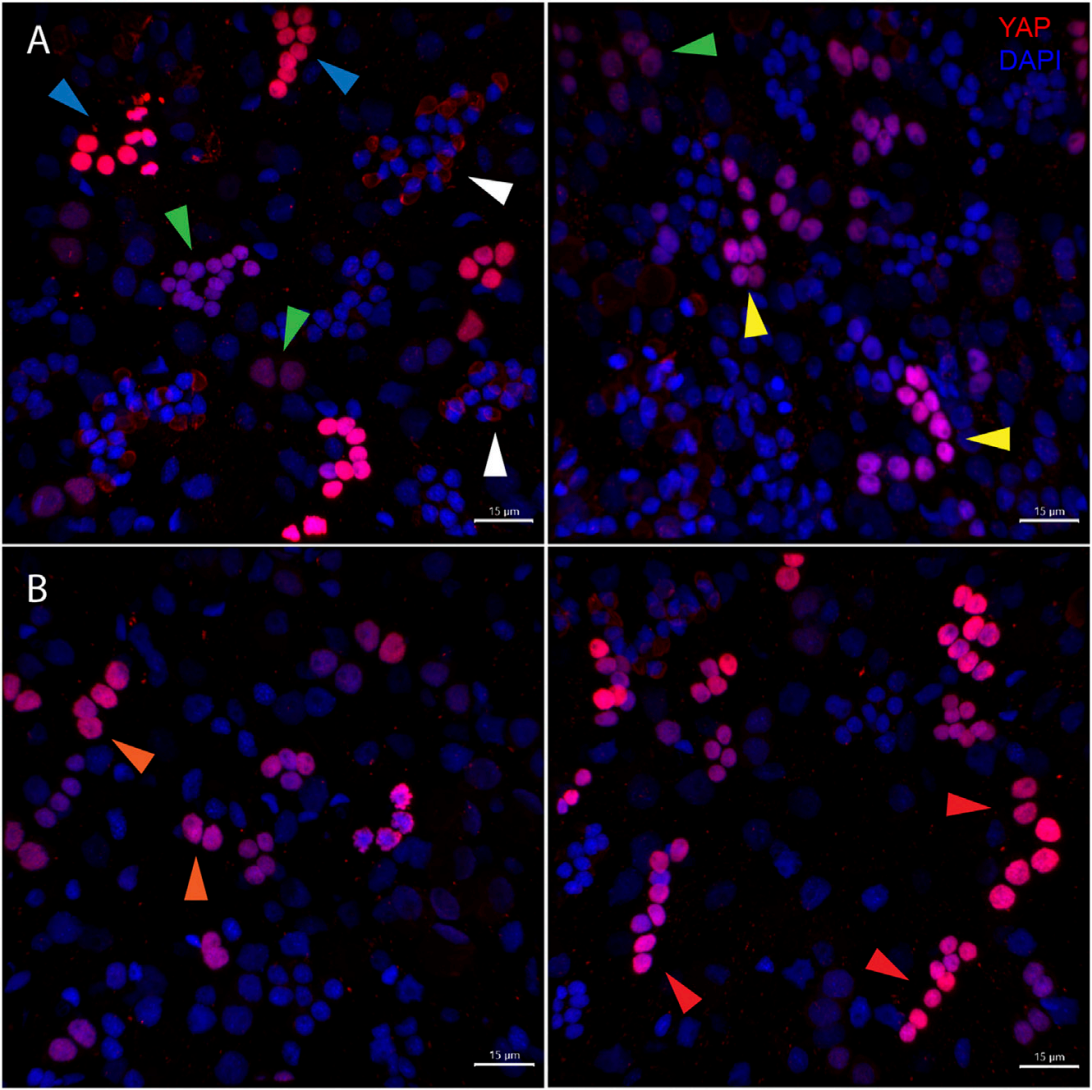
Types of *Hvul*_YAP expressing cells in *Hydra*. Immunofluorescence assay of *Hvul*_YAP performed using anti-CheYki antibody on macerated cells at 60×. **(A)** This panel shows cell types based on signal intensity or YAP expression level in cells. Blue arrow represents cells with high YAP expression, yellow arrow represents cells with medium YAP expression, cells with a green arrow represents low YAP expression. White arrow indicates extra-nuclear staining in nematocysts. **(B)** This panel depicts cell types based on the cellular arrangement. Orange arrows represent cells with duplet or quadruplet arrangement and red arrow represents cells arranged linearly. Red: YAP & Blue: Nucleus (Magenta indicates merged image). Immunofluorescence assay using the anti- *Hvul*_YAP antibody of macerated cells at 60×. The red fluorescent dye shows Alexa 594 staining of YAP and the blue dye shows DAPI staining of nucleus (Scale bar, 20 µm).

The body column of *Hydra* is uniformly interspersed with YAP expressing cells (Figure 6). These cells can be seen almost exclusively in groups (duplets, quadruplets or more). There were specific patterns of these groups that looked similar to those seen in cryosections of ISH samples (Supplementary Figure S4). The expression was clear for nuclearized YAP and a careful analysis of these cells based on the staining intensity and intercellular distance, as seen in IFA, indicates different subsets of cells. Based on the YAP expression intensity, there seem to be cells exhibiting high expression (Blue arrow), medium expression (yellow arrow) and low expression (green arrow). Based on the cellular clustering, cell types can be divided into duplets or quadruplets (orange arrows), which may be interstitial stem cells undergoing differentiation (also Supplementary Figures S7A, S7A’, S8B). There are also clusters of cells that are arranged into a linear file whose identity is difficult to judge (Figure 6- red arrows). Yellow arrows (Figure 6) indicate clusters of cells that looks like part of a nest of nematoblasts. These nest cells are typically arranged into 8–16 cell clusters. As can be noticed here, these clusters are not completely YAP expressing, and only a subset of these express YAP. This may indicate that these cells are expressing only at certain stages of nematoblast differentiation. Such similar clusters can be observed even in the high-level YAP expressing cells (Figure 6- blue arrows), indicating another subset of nematoblast cells. It can also be noted that many of the cells show a basal level of cytoplasmic YAP localization (Pink arrows- Supplementary Figure S7B’). A separate population of cells shows extra-nuclear staining (white arrow- Figure 6 and Supplementary Figure S7B’). These stains might be non-specific since they are localized in cysts similar to those seen in desmonemes and stenoteles. These results suggest that at least some of the YAP expressing cells have interstitial cell origin. Cell sorting analysis of dissociated cells stained for YAP expressing cells revealed that about 65% of the cells were smaller than 200 µm^2^ in size (Supplementary Figure S8A). Among these, most of the cells were nuclearized (71%), while 25% were exclusively cytoplasmically localized (Supplementary Figure S8A’). Based on the size and shape, most cells smaller than 200 µm^2^ can be speculated to be exclusively of interstitial stem cell origin (Supplementary Figures S8A, S8B). The cells larger than 200 µm^2^ in size are mainly epithelial cells (Supplementary Figure S8B’). Identifying the cell types belonging to either endodermal or ectodermal from these proved to be difficult due to rounding of cells after enzymatic dissociation of the tissue. While the inner hypostome (area immediate around the mouth) and the tentacles are virtually devoid of YAP expressing cells, we find a few non-clustered YAP expressing cells at the region interstitial to the tentacle bases and the outer hypostome (Figure 5 and Supplementary Figure S9B). Such an expression pattern may indicate a role of YAP in tissue compartment-boundary regulation for hypostomal and tentacle development and/or maintenance of gene networks in *Hydra*. Such a role of Yki (YAP) has been recently proposed in *Drosophila* in wing imaginal disc development by regulating the expression of Hox genes and Hedgehog signaling (Bairzin et al., 2020).

#### YAP Expressing Cells Are Recruited to Newly Developing Buds but Are Excluded From the Hypostomal Region Upon Initiation of Differentiation

**FIGURE 7:**
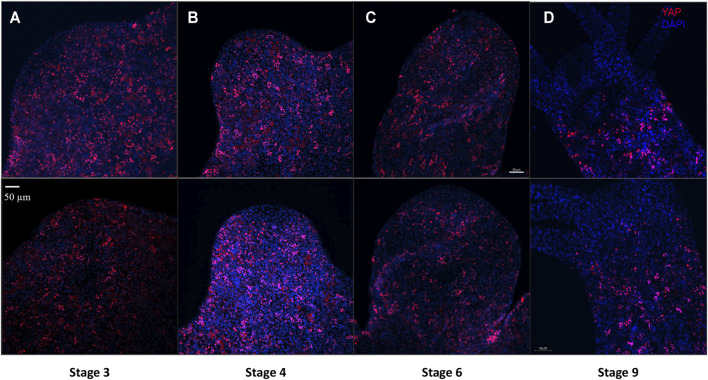
YAP positive cells are recruited early to the bud tip and are excluded from the region which are terminally differentiated in the late stages of bud development. Immunofluorescence assay of *Hvul*_YAP performed using anti-CheYki antibody for different budding stages (represented by two polyps for depicting each stage) of *Hydra* showing recruitment of YAP positive cells to the budding tip. **(A)** Stage 3 shows early recruitment of YAP positive cells to the emerging bud with non-clustered cells at the distal tip with slight depletion at tip of the bud. **(B)** at Stage 4, depletion of the YAP expressing is more prominent which gets further exaggerated at **(C)** Stage 6 and **(D)** Stage 9. Red: YAP & Blue: DAPI (Scale bar, 50 μm, *N* = 3 per stage).

Cellular dynamics of YAP expressing cells during *Hydra* bud development was studied using immunofluorescence assay. Buds at different points of bud development from early to late stages were observed (Stage 3, 4, 6 and 9). It was clear that the YAP expressing cells moved into the early bud with an expression pattern similar to that found in the body column. Such a pattern is persistent throughout the budding stages in the body column of the newly developed bud. The most interesting changes happening to the YAP expressing cells in a bud is at the hypostomal region. The YAP expressing cells near the distal bud tip were non-clustered compared to the rest of the lower bud region. At stage 3, the bud-tip where the head organizer has been set to establish the new body axis for bud, YAP expressing cells seem to be depleted (Figure 7A). This pattern is even more conspicuous from stage 4 onwards (Figures 7B–D and Supplementary Figure S9A). From stage 9 onwards, the expression pattern similar to the adult *Hydra* is established where we see non-clustered YAP expressing cells seen sparsely at the boundaries between the hypostome and the tentacle base (Figure 7D and Supplementary Figure S9B). The appearance of non-clustered cells in these regions may indicate a different sub-type of YAP expressing cells having a role in head organizer maintenance in *Hydra*. Another interesting point is that YAP expressing cells are completely depleted at the basal disk (Figure 5), hypostome and tentacles. This observation may indicate a critical antagonistic role of YAP signaling in tissues with terminally differentiated cells. The lack of YAP expressing cells even at the early developmental stages of tentacle development in a new bud (Supplementary Figure S9B) and at the Adult-bud boundary where the basal disk will form (Supplementary Figure S9C) further suggests the possibility of Hippo pathway in cell differentiation.

#### Actively Proliferating Cells Co-express YAP

**FIGURE 8:**
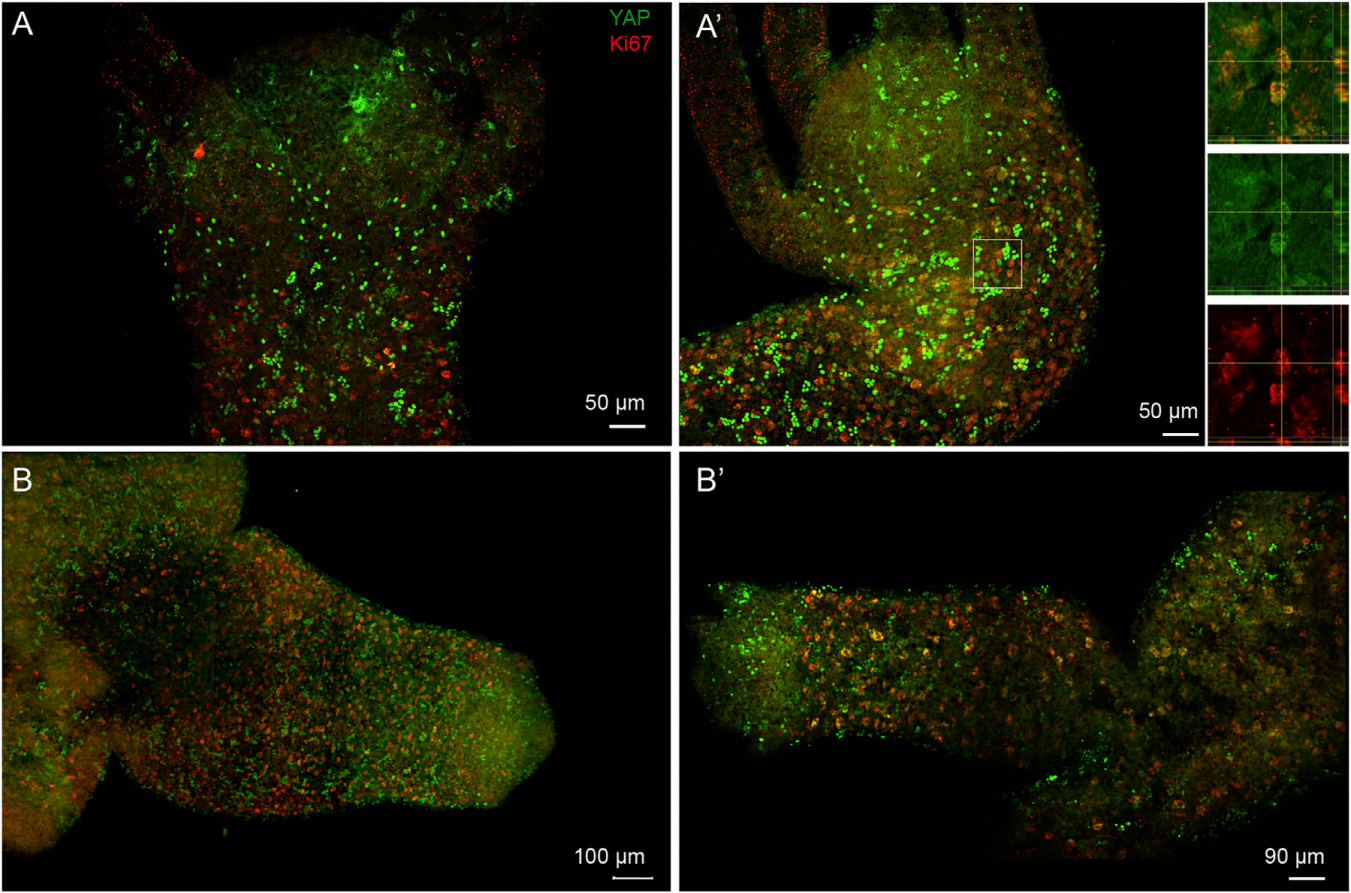
Subset of nuclearized YAP positive cells are actively proliferating cells and are actively recruited to the developing bud. Immunofluorescence assay of Hvul_YAP performed using anti-CheYki antibody and proliferating cell population using Ki67 marker showing colocalization of a subset of YAP positive cells. The images shown here are maximum intensity projections. **(A**,**A’)** This panel shows the head and shoulder region of the polyp showing brightly stained nuclearized YAP expressing cells are not actively proliferating. The individual cells expressing YAP in the outer hypostomal region are also non-proliferative. The white square in figure **(A′)** is enlarged in the insets shown on the right of the figure. The top inset shows the tissue from a X-Y plane in the centre with orthogonal slices of Y-Z and X-Z plane indicated in right and below respectively. The region of interest wherein one of the cells showing colocalization is confirmed on the X-Y-Z axes. Middle inset shows the same image in green channel (YAP) and lower inset shows the red channel (Ki67). **(B**,**B’)** The budding polyps showing active incorporation of proliferative cells into the developing bud. These cells co-stain with Hvul_YAP suggesting their colocalization. Panel **(B)** clearly shows small and dense localization of actively proliferating cells being recruited into the newly forming bud in early stages. The panel **(B′)** shows a mature bud where the tentacles have started forming and the active recruitment of cells into the bud body has reduced. Hence, the Ki67 expressing cells are larger in size and less dense. (*N* = 3), Red: Ki67 & Green: YAP. (Scale bar: A and A’, 50 μm, B, 100 µm and B’, 90 µm).

Co-immunostaining for YAP with cell proliferation marker Ki67 was performed to assay if the reported role of YAP in cell proliferation in more complex organisms like Drosophila, mouse etc., is conserved in *Hydra* as well. The immunostaining by Ki67 revealed expression exclusively in the body column and a complete lack in the regions where the cells are terminally differentiated (hypostome, basal disk or tentacles) (Figure 8). The co-staining experiment revealed that most of the proliferating cells in the polyp also co-express YAP (Figure 8A’ (inset) and Supplementary Figure S10). Further, the subset of YAP expressing cells with a high expression level was not actively proliferating and may indicate being in an arrested phase or are actively differentiating (Figures 8A, 8A’). The non-clustered cells observed in the outer region of the hypostome in adult *Hydra* and the budding *Hydra* both lack any Ki67 co-staining and hence are non-proliferating cells. The actively proliferating cells exhibit loosely packed nuclei and low staining of YAP in the nucleus. In budding polyps, due to sustained induction of actively proliferating cells from the polyp to the bud, we observed a very high density of Ki67 expressing cells (Figures 8B, 8B’ and Supplementary Figure S10). YAP co-staining in actively proliferating cells reinforces its conserved role in basal metazoan such as *Hydra*. A similar observation has been previously reported in *Clytia*, a fellow cnidarian (Coste et al., 2016).

### Verteporfin Treatment Increases the Rate of Budding in *Hydra*

**FIGURE 9:**
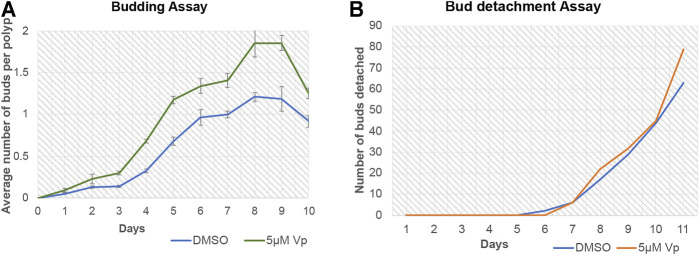
Disruption of YAP-TEAD interaction in *Hydra* leads to an increase in budding kinetics. The YAP-TEAD interaction in *Hydra* was targeted using verteporfin (Vp) and the budding rate of the polyps were assayed against DMSO treated control polyps by calculating average buds per *Hydra* for the period of 10 days post treatment. **(A)** Vp treatment (5 µM) led to significant increase in the budding rate in Vp treated polyps as compared to the DMSO treated control polyps from day 3–10 (Refer Supplementary Table S2 for statistical data). 48 polyps were scored per set per experiment and total of three biological replicates were performed for each set. **(B)** Bud detachment assay was performed by calculating cumulative buds detached on each day. This experiment showed no change is Vp vs DMSO treated polyps indicating that increased budding rate is a function of cell proliferation and not bud detachment rate.

Verteporfin (Vp) is a benzoporphyrin derivative small inhibitor routinely used as a potent inhibitor for YAP-TEAD interaction (Brodowska et al., 2014). Vp binds to YAP and prevents it from interacting with TEAD. A budding assay was performed to assess the effects of Vp on the budding of *Hydra* polyps throughout 10 days of treatment. Vp treatment led to an increase in the average number of buds per polyps (the total number of buds from a set of 48 polyps were calculated and then divided by 48 to obtain the value) (Figure 9A). To confirm that the increased buds per polyp were not due to delayed rate of bud detachment, an assay was performed to quantitate the cumulative number of fallen buds (detached). As shown in Figure 9B, the detachment rate did not change drastically in the presence of Vp. These observations indicate that Vp treatment causes the polyps to produce more new buds than the control. Since the budding rate is indicative of the rate of cell proliferation and maintenance of steady-state, Vp treatment is also indicative of increased cell proliferation.

## Discussion

Detailed characterization of the Hippo pathway and its components in pre-bilaterians has been extremely sparse. There have been few studies reporting the presence of Hippo homologs in these primitive organisms. *Capsaspora owczarzaki*, a single-celled eukaryote, is the most primitive organism predicted to have a complete set of functional core Hippo pathway homologs indicative of a holozoan origin of the functional pathway (Sebé-Pedrós et al., 2012). Another study confirmed the presence of Hippo pathway components in a ctenophore species: *Pleurobrachia pileus,* and a cnidarian species *Clytia hemispherica* (Coste et al., 2016). While this study reported an absence of Yki in ctenophores, it showed that the Yki in *Clytia* is conserved to regulate cell proliferation and growth. In this study, we have, for the first time, identified and characterized a complete set of core Hippo pathway components in *Hydra vulgaris* through bioinformatic analysis and cloning. The current phylogenetic analysis is congruent with previous reports that *Nematostella vectensis* homologue is more similar to complex vertebrates (Hilman and Gat, 2011). In fact, all the Cnidarian homologues exhibit higher similarity with the chordate YAP sequences. This suggests that YAP sequences evolved close to the emergence of the chordate homologs and might exhibit similar properties observed in these organisms. A recent study proposed such a possibility where a detailed sequence analysis of YAP homologs in model organisms across animal phyla was performed (Elbediwy and Thompson, 2018). The study indicated that Deuterostomes and the more basal cnidarian might exhibit similar regulation *via* integrins present in the basal region of the cells due to their inherent stratified/pseudostratified epithelial structure. On the other hand, the protostome YAP may be exclusively regulated *via* apical signals due to their monolayer columnar epithelial organization (Elbediwy and Thompson, 2018). In our study, the domain organization analysis indicates divergence in the N-terminal homology domain of YAP (FAM181) in different lineages. This suggests the clade-specific role of the FAM181 region, probably in the interactions with TEAD like or other proteins. This further indicates taxon-specific modification took place in the FAM181 region and might play lineage-specific functions. We show that the Hippo pathway components are more or less uniformly expressed throughout the polyp tissues barring a few regions in a gene-specific manner like budding zone, early buds, extremities of the polyps such as tentacle tips or basal disks. Considering the studies in bilaterians indicating that these components are all tightly controlled to regulate the cell cycle and cell differentiation, it can be easily seen why these genes are expressed uniformly in all tissues. Since the extremities of the polyps are terminally differentiated, they probably do not need these genes for the functions mentioned above and are already set to perform their designated functions without any change. The analysis of amino acid sequences of these genes to predict the secondary structure and 3D tertiary protein models have also given us some insightful results. Domain architecture of all the Hippo pathway proteins shows that their architecture is well conserved in Cnidaria, which confirms the pathway’s ancient establishment and evolution in the basal metazoans. The 3D modelling of YAP’s TBD and TEAD’s YBD in *Hydra* using the published crystal structure of their Human homolog predicts a similar interaction capability of YAP and TEAD in *Hydra*. Our analysis revealed that the YAP-TEAD complex is highly stable in *Hydra*. This raises the possibility that the YAP-TEAD interaction was robust in primitive metazoans, and as the signaling pathway evolved, the stability of the complex was presumably partially compromised to accommodate the promiscuous nature of YAP in more complex organisms. This indirectly indicates that the functions of the Hippo pathway or YAP signaling reported in bilaterians may have been established as early as in Cnidarians and hence may have played in developing important characteristics of multicellular organisms like cell-type divergence, body-axis development, germ-layer differentiation etc.

In *Clytia*, it was found that Yki was nuclearized at the tentacle base where there are highly proliferating cells, while they are inhibited in the tentacles where the cells are differentiated (Coste et al., 2016). Using the antibodies used in the same study, we were able to study the protein-level expression of YAP in *Hydra*. We find that even though *Hvul_yap* is expressed uniformly throughout the polyp, only a few cells have *Hvul*_YAP in the “active form” (nuclearized). We find that these nuclearized YAP are more or less uniformly spread throughout the polyp. YAP expression is almost absent or not nuclearized in the terminally differentiated regions, including the tentacles, hypostome or basal disk. An interesting observation is the presence of YAP expressing cells at the tentacle base forming a circle (Figure 5 and Supplementary Figure S7B). This can be considered homologous to the expression pattern seen in *Clytia*, which may be speculated as necessary for the terminal differentiation of cells while crossing the body column-tentacle boundary. Another possibility can be the mechanical activation due to physical stress experienced at the tentacle base due to the movement of tentacles or anatomical constraints. Most of these cells in the body column can be seen in groups or colonies. Cellular features and arrangements of YAP positive cells are indicative of interstitial stem cell origin. Cells like desmonemes and stenoteles are mechano-sensitive, and YAP may regulate their development and function. Based on the quantitative flow cytometry, we find that most of the population with nuclearized YAP are interstitial stem cell-based. This observation supports the proposed mechanism of the ancient role of active YAP signaling in basal cells (interstitial cells in *Hydra*), which lack a proper apical domain due to pseudo-stratification (Elbediwy and Thompson, 2018). Another interesting observation is the presence of a non-clustered group of cells in the outer hypostomal region (Supplementary Figure S9B). Such an expression pattern raises many interesting possibilities. It is reported that the ectodermal cells in the hypostome are maintained separately from the gastric region (Dübel et al., 1987; Dübel, 1989). The inner hypostomal ring consists exclusively of terminally differentiated cells (Dübel, 1989). The stationary region in the hypostome (the outer hypostomal ring) contains a population of the ectodermal epithelial cells that retains its proliferative potential, which contributes exclusively to the cell types in the entire hypostomal region. Once the hypostome is specified, there are no contributions from the gastric ectoderm towards hypostomal cells unless the hypostome is lost upon amputation. A unique population of YAP expressing cells (non-clustered cells) in the outer hypostomal region and not at the inner region may indicate the possibility of differential mechanical properties attributing specialized functions of these cells. An identical population of cells can be found during early bud development. These observations raise the possibility of these cells having a crucial role in establishing and maintaining the head organizer. This regulation may well be activated biochemically with pre-existing cues. Previously, one of the variants of *brachyury* (Hy*Bra2*) was shown to express early during the bud formation (Bielen et al., 2007). This study also showed that the same *brachyury* variant was also expressed early during head regeneration in *Hydra* (8 h post-amputation). Interestingly, the expression pattern of *HyBra* is exclusively at the hypostomal region encompassing both the outer and inner hypostome (Technau and Bode, 1999; Bielen et al., 2007). *Bra* is known to be a direct responder to the consolidation of Wnt/β-catenin signaling (Yamaguchi et al., 1999). *HyBra* has also been implicated in the establishment of the head organizer in *Hydra* (Technau and Bode, 1999). Hence, this may mean that appearance of *HyBra* may coincide with the true setting up of the head organizer. This raises the enticing prospect of YAP expressing cells at the outer hypostome region to restrict the head organizer-related function of Brachyury to the inner hypostome ring by exerting its tissue boundary regulation functions *via* the hedgehog pathway (Bairzin et al., 2020). Taken together, the expression pattern of YAP in developing bud and adult polyp consolidates the possibility of YAP in the establishment and maintenance of the head organizer function in *Hydra*.

Our study reinforces the well-established role of YAP in cell proliferation, even in basal metazoans such as *Hydra*. Our results support the observation reported in *Clytia*, where CheYki was shown to be nuclearized in the proliferating zones of the animal (Coste et al., 2016). With most of the actively proliferating cells in the gastric region of *Hydra* polyp showing nuclearized YAP and these cells being recruited to actively growing buds in high density, together strongly suggest an important role of YAP in cell proliferation in *Hydra.* Interestingly, we also note that disruption of YAP-TEAD interaction led to an increase in the budding rate in *Hydra*, which can be attested to an increased cell proliferation rate. A similar observation was shown in *Schmidtea mediterranea* (Lin and Pearson, 2014), wherein it was reported that Yki in *S. mediterranea* is required to restrict stem cell proliferation and regulate organ homeostasis. Yki knockdown showed a hyper-proliferation and increased *wnt* expression*.* Such a phenotype is completely opposite of the under-proliferation phenotype reported in other well-studied model systems except for mammalian intestine stem cell models (Lin and Pearson, 2014). The same group later reported that a similar restrictive function of Yki comes into play during regeneration, where it is required to help remodel and scale according to the requirement of regenerating fragments (Lin and Pearson, 2017). Taken together, our data indicate an ancient function of YAP and WNT in cell proliferation and tissue homeostasis with restrictive control on both in cells that lack proper apical signaling.

*Hydra* is considered to be immortal due to its capability to maintain a steady-state of cell death and cell proliferation. This study shows that the Hippo pathway is an important signaling pathway capable of regulating the cellular differentiation, proliferation and budding in *Hydra*. YAP signaling may play an important role in maintaining tissue homeostasis in the Hydra in such a context. A more in-depth study of YAP signaling under these contexts might reveal interesting insights into the evolution of the functions associated with complex organisms. YAP can act as a mechanotransducer and has been shown to regulate various morphogenetic and developmental functions. This aspect of YAP is only starting to be fully understood and have been poorly studied in basal metazoans to understand its origins. A detailed study in *Hydra* to understand the same will shed light on the fundamental aspects of how tissue mechanics plays a role in regulating cell function.

## Data Availability

The datasets for this study can be found in Supplementary Tables S1–S4. This includes the protein sequences used for phylogenetic analysis (Supplementary Table S1), Raw data and statistical analysis used for budding assay (Supplementary Table S2), BLAST hit list of proteins from Hydra against CheYki peptide (Supplementary Table S3)and lastly, the mRNA sequences and protein sequences of the Hydra Hippo pathway core homologs in separate tabs of the excel sheet (Supplementary Table S4).
