# Supplementary material for: Identification of Components of the Hippo Pathway in Hydra and Potential Role of YAP in Cell Division and Differentiation
Source: Front Genet. 2021 Oct 6;12:676182. doi: 10.3389/fgene.2021.676182 (PMC8526868; doi:10.3389/fgene.2021.676182)
Supplement: Supplementary file 5 [file DataSheet1.docx]

Supplementary Figures

**
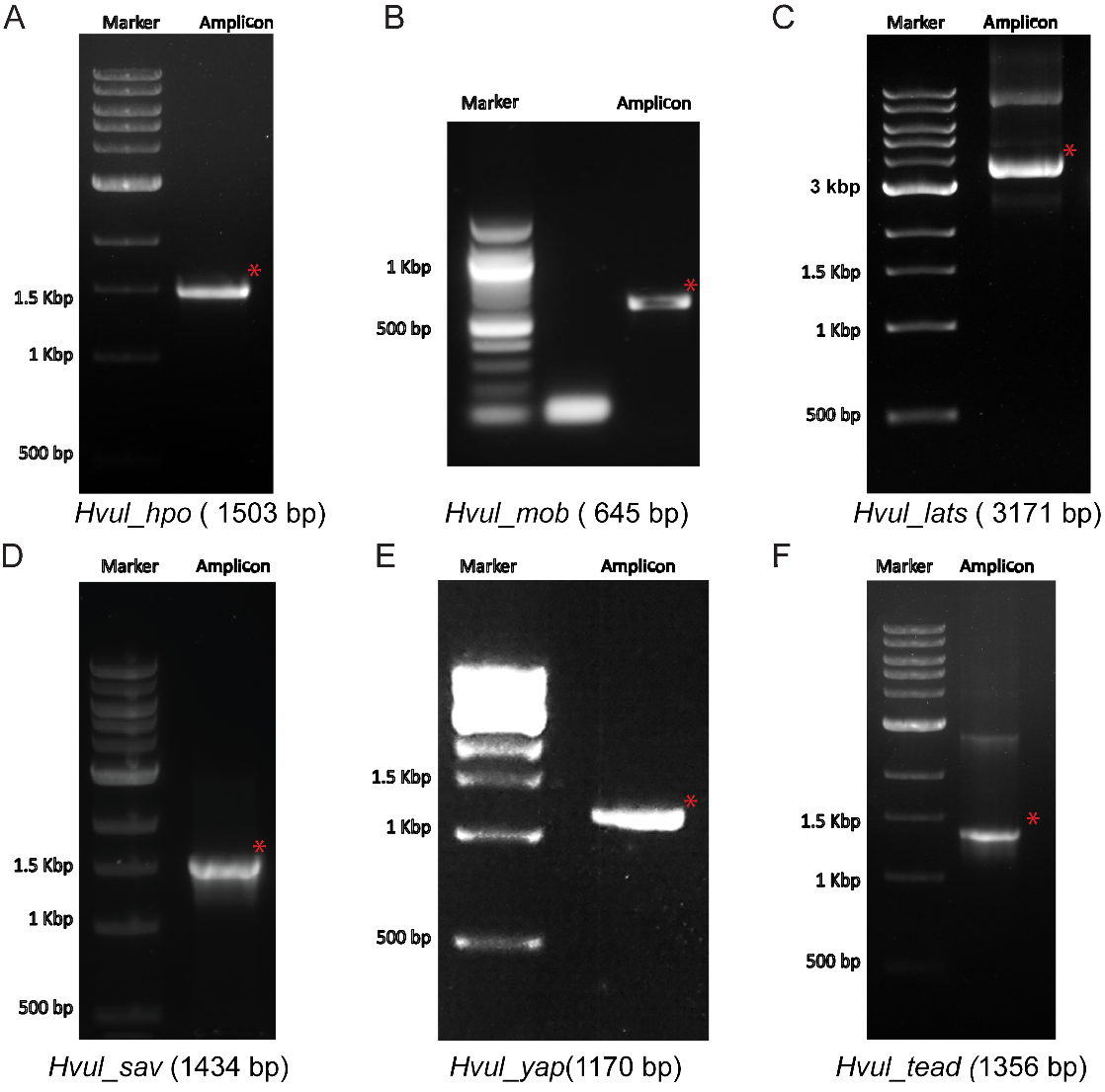
**

**Supplementary Figure 1.** **Validation of the Hippo Pathway homologs in *Hydra* by PCR mediated amplification using the predicted sequence**. **(A)** *Hvul*_Hpo was amplified at 1503 bp. **(B)** *Hvul*_Mob was amplified at 645 bp. **(C)** *Hvul*_LATS was amplified at 1170 bp. **(D)** *Hvul*_SAV was amplified at 1434 bp. **(E)** *Hvul*_YAP was amplified at 1170 bp. **(F)** *Hvul*_TEAD was amplified at 1356 bp.


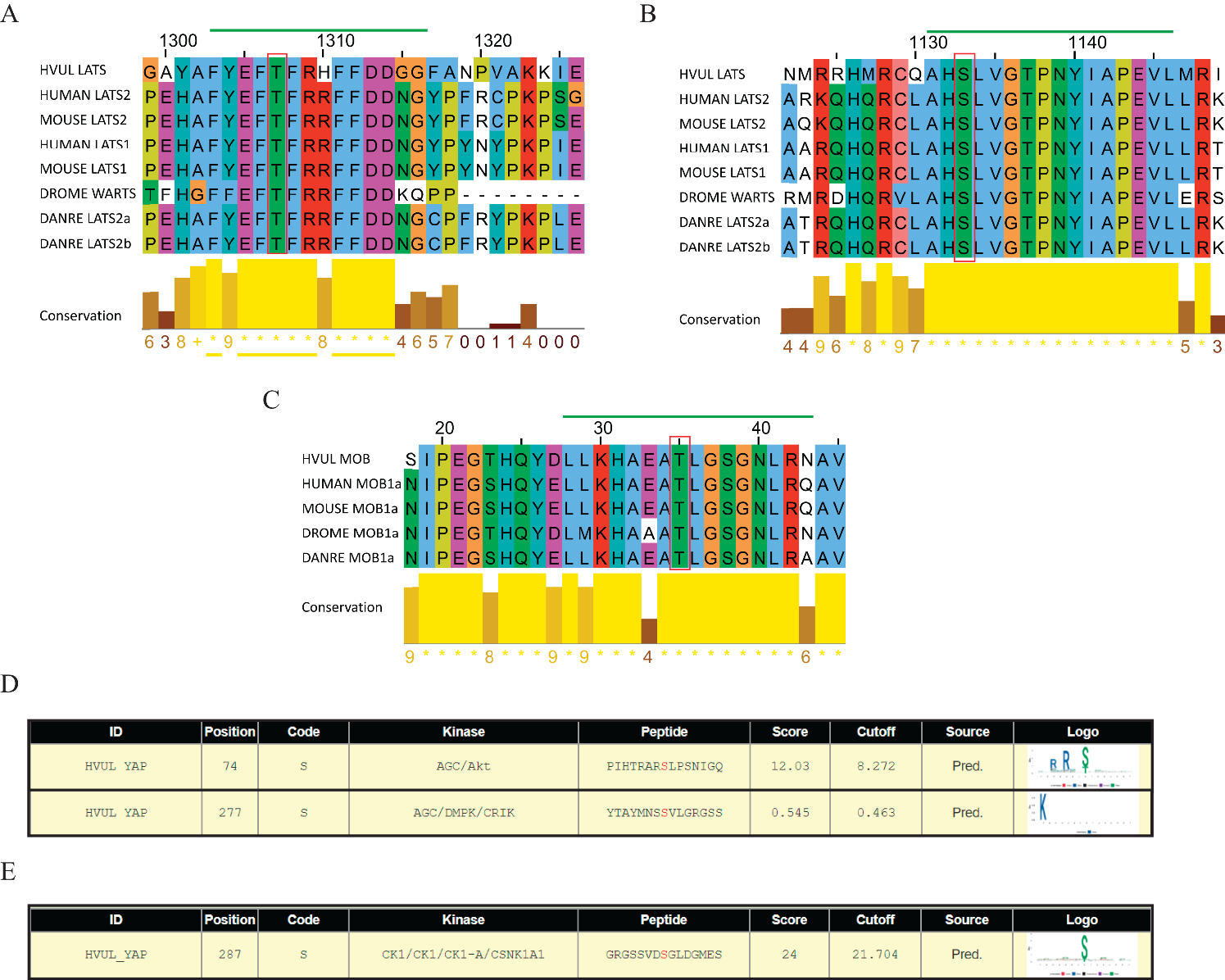


**Supplementary Figure 2.** **Motif analysis of LATS, MOB and YAP. (A)** Conserved hydrophobic motif of the LATS with threonine (T1079) required for the activation of LATS by HIPPO phosphorylation highlighted by red rectangle. **(B)** The auto-activation T-loop in LATS is 100 % conserved across the species (S909 is indicated by red rectangle). **(C)** Highly conserved motif in MOB required for regulation of MST-SAV-LATS-MOB complex and LATS auto-activation (T35 is indicated by the red rectangle. Colour scheme for amino acid residues: Blue- Hydrophobic, Red- Positive charge, Magenta- Negative charge, Green- Polar & Cyan- Aromatic. The green line at the top of the figures A, B and C indicates the motif of interest. **(D)** Predicted LATS phosphorylation site in *Hvul*_YAP at S74 (homologous to the mammalian S127) and S276 (homologous to the mammalian S381). **(E)** Phosphodegron motif predicted in *Hvul*_YAP immediately downstream to the S276 (S381 in humans).


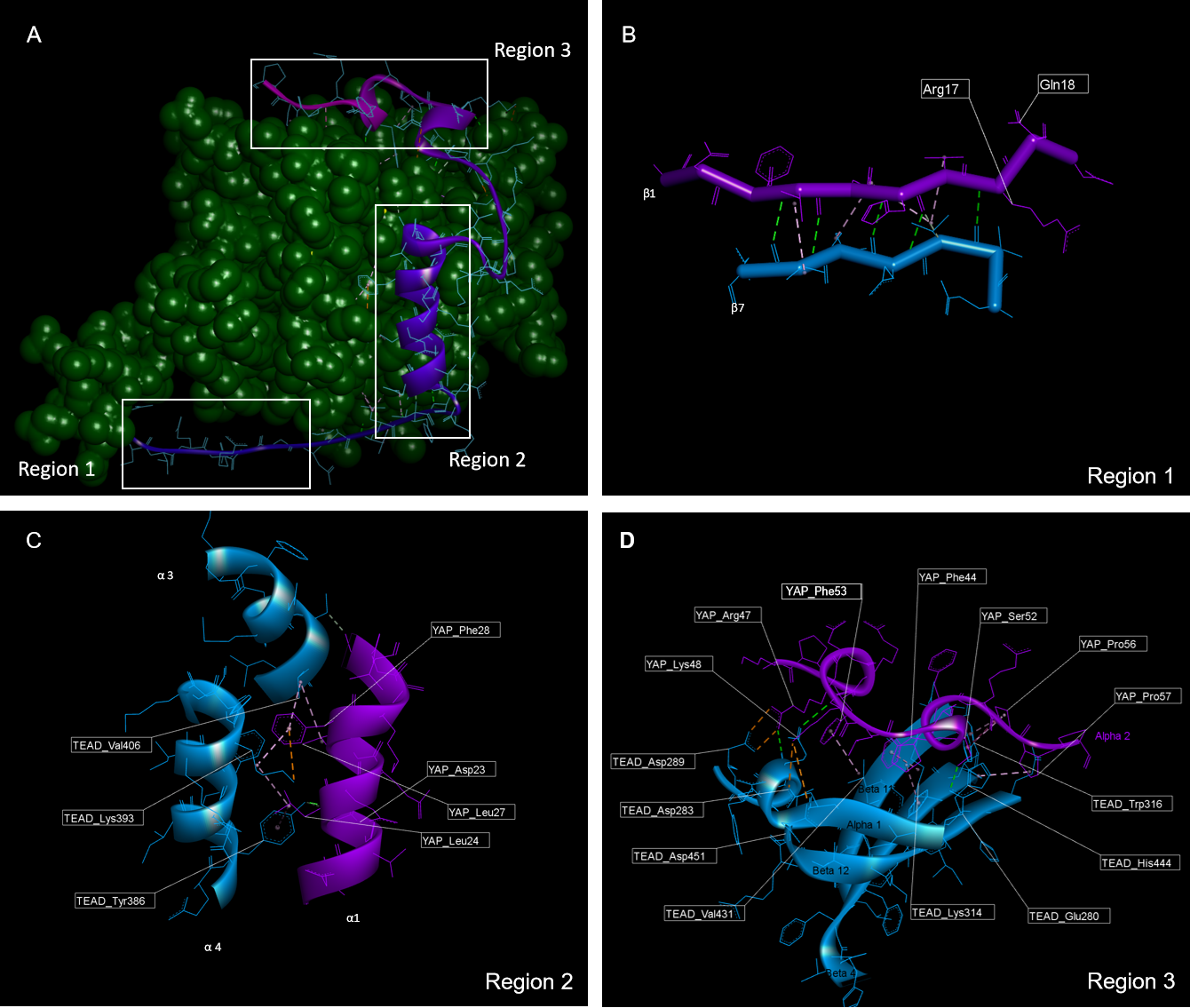


**Supplementary Figure 3.** **3D structural model of YBD and TBD in the *Hydra* YAP-TEAD complex.** The 3D structure of the YBD and TBD of *Hvul*_YAP and *Hvul*_TEAD was modelled on human PDB structural model of YBD-TBD (4RE1) using MODELLER software. **(A)** The modeled YBD-TBD complex of *Hydra* showing three different regions- Region 1, Region 2 & Region 3 of TBD (purple) interacting with the globular YBD (green). **(B)** The Region 1 interface forming an anti-parallel β sheet consisting of TBD β1 and YBD β7 strands of *Hydra* interacts with only six hydrogen bonds (green dotted lines) due to the presence of Gln18 in β1 instead of Gly59 found in humans. **(C)** Region 2 has the α1 helix of the TBD (amino acids 20-32) fitting right into the binding groove of the YBD formed by the α3 and α4 helices of the YBD (amino acids 385-409). This interaction mainly consists of Leu24, Leu27and Phe28 from TBD and Try386, Lys393 and Val406 of YBD (pink dotted lines).  **(D)** The region 3 in *Hydra*, has hydrophobic side chains of the TBD – Phe44 (Met86 in humans), Leu49, Pro50 and Phe53 forming extensive van der Waals interactions with the YBD of TEAD at Glu280, Ala281, Ile282, Gln286, Ile287, Leu312, Leu316, Val431, His444 & Phe446. The interface is further strengthened by multiple hydrogen bonds (indicated in green dotted lines). A predicted ability to form two salt bridges (orange dotted lines) in this region in *Hydra* model as compared to just one in the humans- TBD_Arg47:YBD_Asp289:YBD_Asp289 and TBD_Lys48:YBD_Asp283:YBD_Asp451.


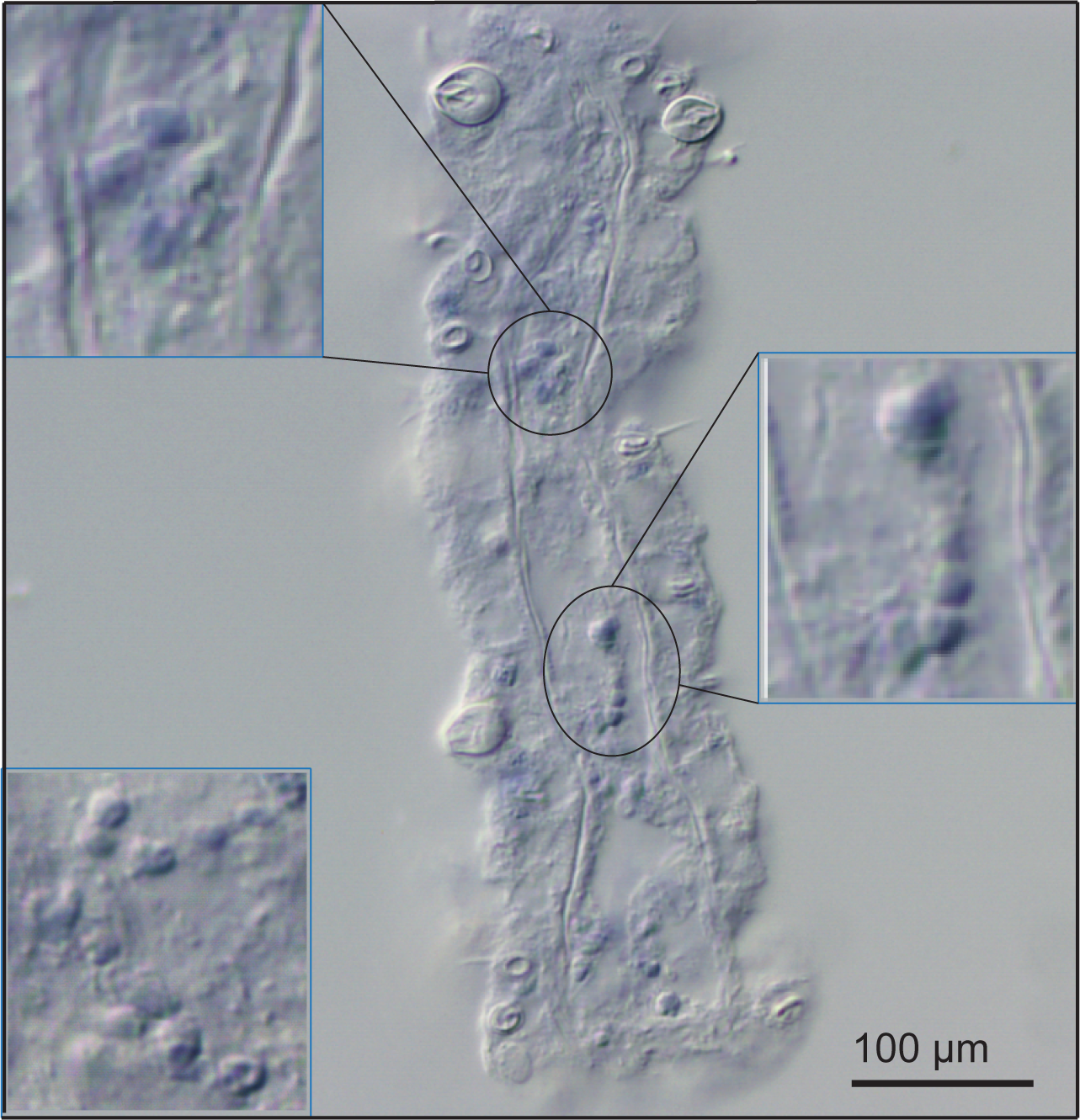


**Supplementary Figure 4.** **Cryosection of YAP WISH at tentacle region showing cell-type specific expression**. Cryosectioning of the polyps which were stained for YAP expression by WISH were done by embedding them in PVP. These embedded polyps were cut into 25 µm sections using cryotome and then imaged. The image reveals cells in doublets, quadruplets and groups of cells among other stained cells indicating their interstitial stem cell origin, plausibly nematoblast and nests of nematoblasts. (insets shows zoomed-in areas indicted by black circles)


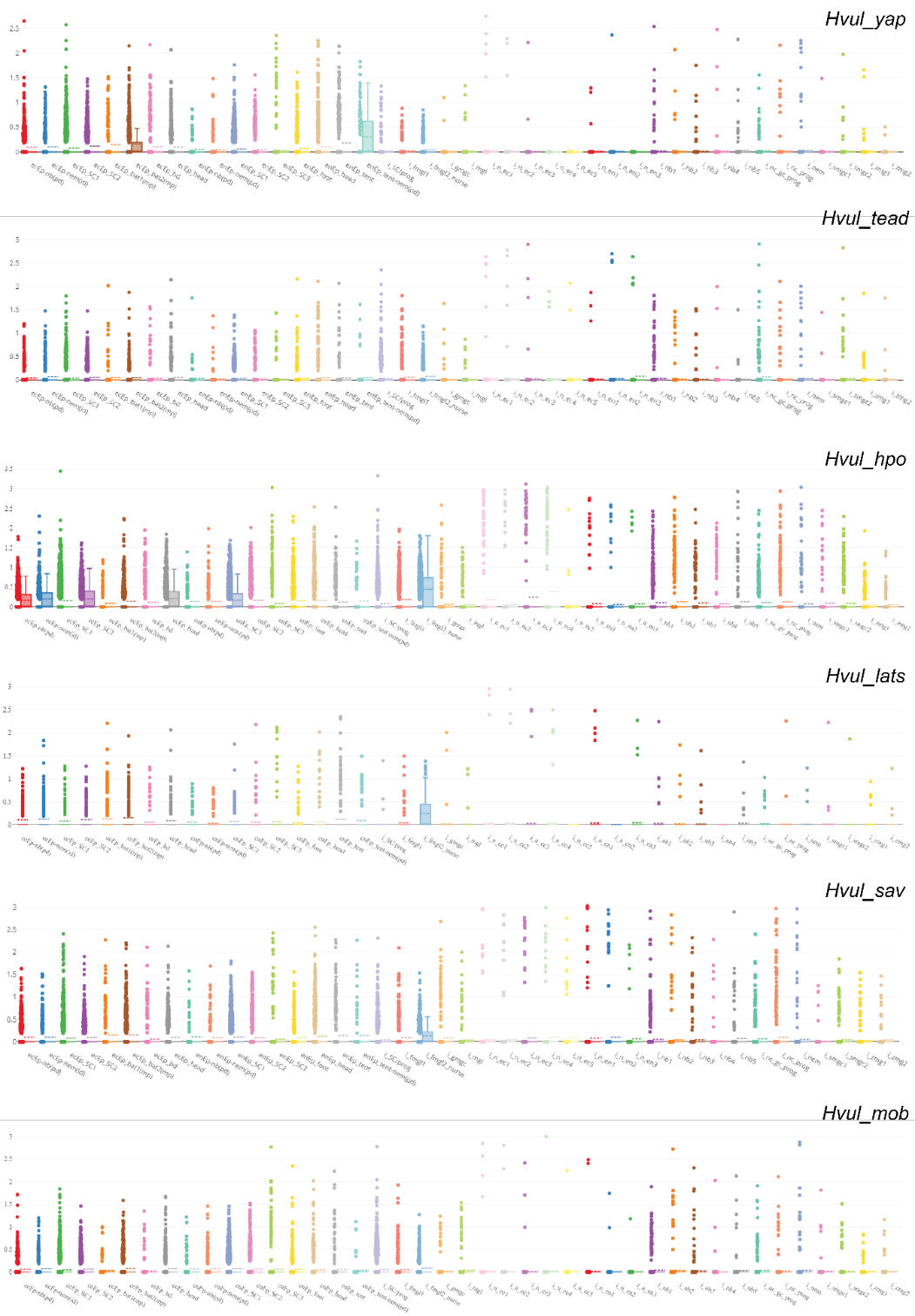


**Supplementary Figure 5.** **Differential expression of the Hippo pathway components** **in various cell types.** The differential expression of six *Hydra* Hippo genes and was examined using the Single Cell Portal. The expression pattern for Hvul_yap, Hvul_tead, Hvul_hpo, Hvul_lats, Hvul_sav & Hvul_mob is enlisted respectively from top to bottom of the figure. Cluster label abbreviation key: bat: battery cell, bd: basal disk, db: doublet cluster, ec: ectoderm, ecEP: ectodermal epithelial cell, en: endoderm, enEP: endodermal epithelial cell, fmgl: female germ-line, gc: gland cell, gmgc: granular mucous gland cell, i: cell of the interstitial lineage, id: integration doublet, mgl: male germline, mp: multiplet, nb: nematoblast, n: neuronal cell, nem: nematocyte, pd: suspected phagocytosis doublet, prog: progenitor, SC: stem cell, smgc: spumous mucous gland cell, tent: tentacle, zmg: zymogen gland cell.


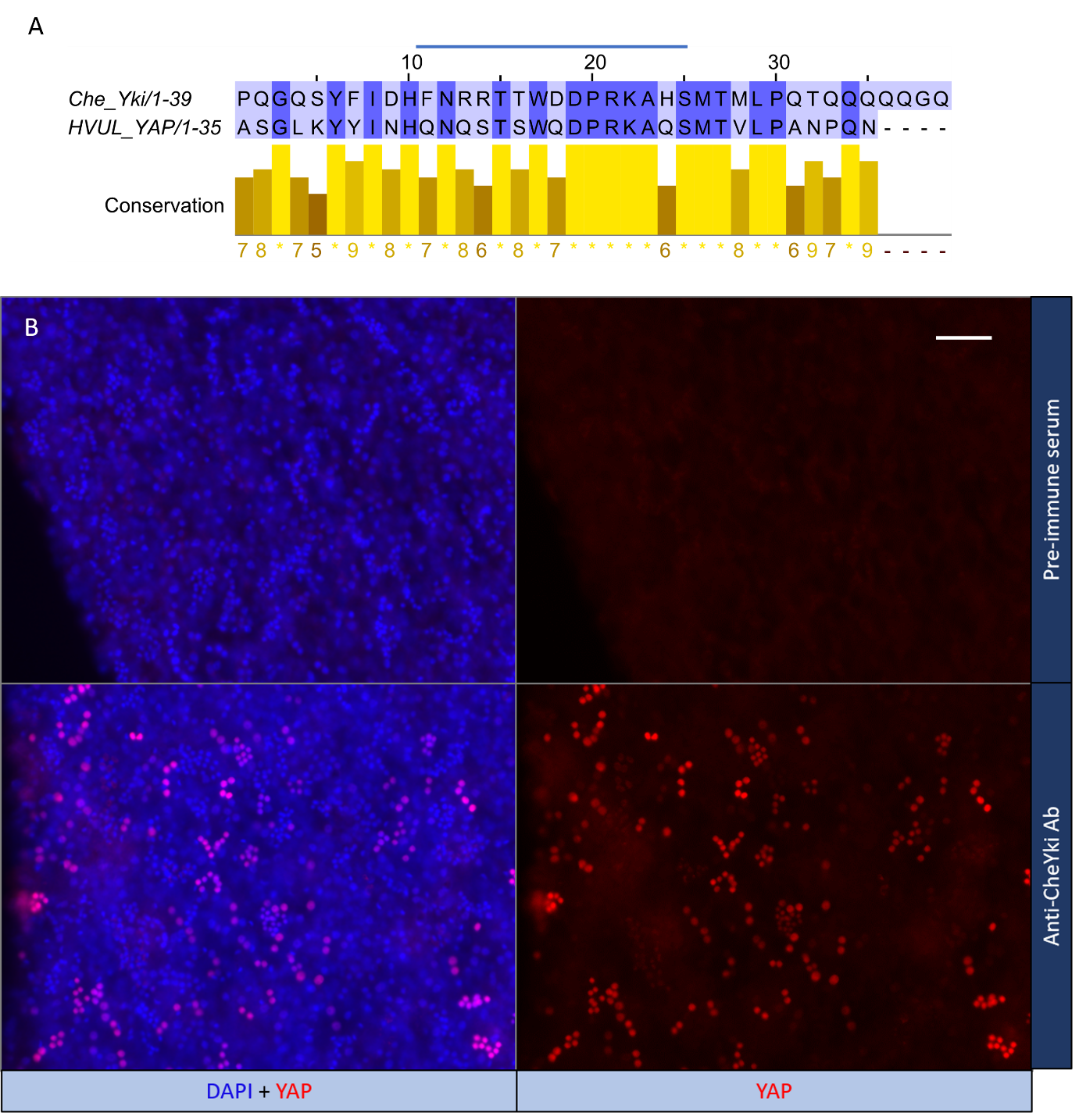


**Supplementary Figure 6.** **Validation of CheYki antibody in *Hydra* by immunofluorescence assay (IFA). (A)** A peptide-specific (immunogen) alignment between *Clytia* Yki and *Hydra* YAP showing a 66% similarity and a 60 % identity at motif. **(B)** IFA in *Hydra* polyps using CheYki antibody showing staining pattern of *Hvul*_YAP against the negative control (pre-immune serum). The IFA yielded a robust signal for CheYki antibody as compared to the negative control. Red: YAP & Blue: DAPI. (Scale bar = 50 µm)


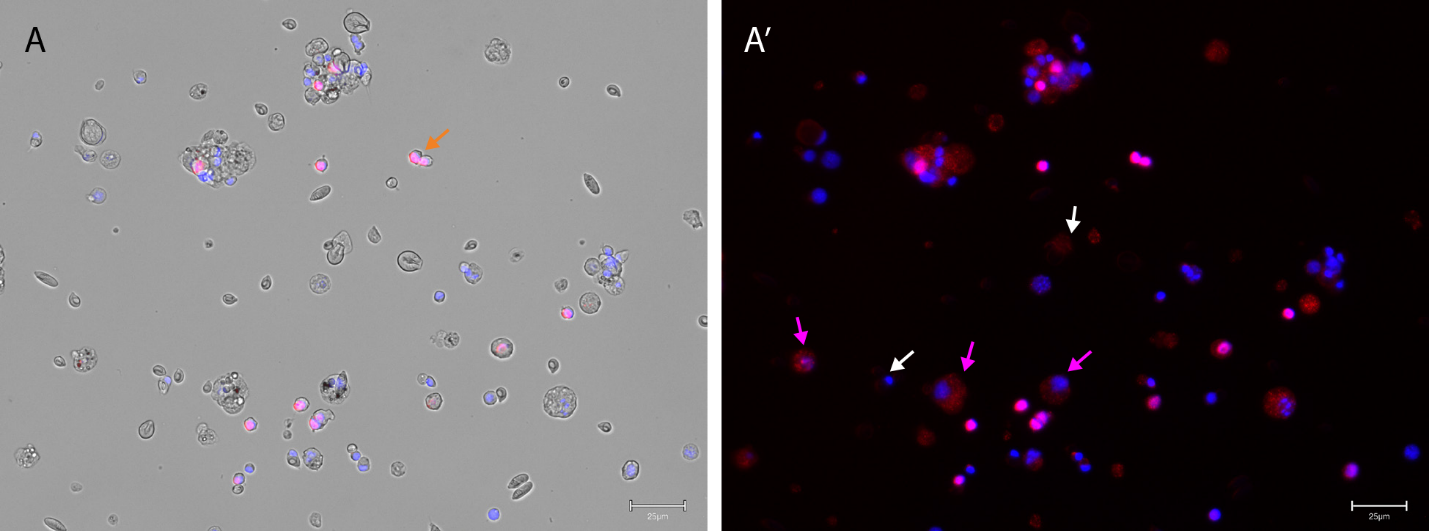


**Supplementary Figure 7.** **Identification of types of *Hvul*_YAP expressing cells in *Hydra* by immunofluorescence assay (IFA) of dissociated cells.** Immunofluorescence assay of *Hvul*_YAP performed using anti-CheYki antibody on enzymatically dissociated cells at 40X.  **(A)** This panel shows cell types based on localization of YAP in cells and cell morphology seen clearly from the brightfield image. Orange arrow indicates nuclearized YAP expressing duplet cells of interstitial stem cell origin. Blue arrow represents cells with high YAP expression, yellow arrow represents cells with medium YAP expression, cells with a green arrow represents low YAP expression. (**A’)**. This panel depicts the same cells as in panel without the brightfield to observe basal expression of YAP in the cytoplasm. White arrow indicates extra-nuclear staining in nematocysts. And pink arrow indicates cells with cytoplasmic localization. Red: YAP & Blue: Nucleus (Magenta indicates merged image). The red fluorescent dye shows Alexa 568 staining of YAP and the blue dye shows DAPI staining of nucleus. (Scale bar: 25 µm).


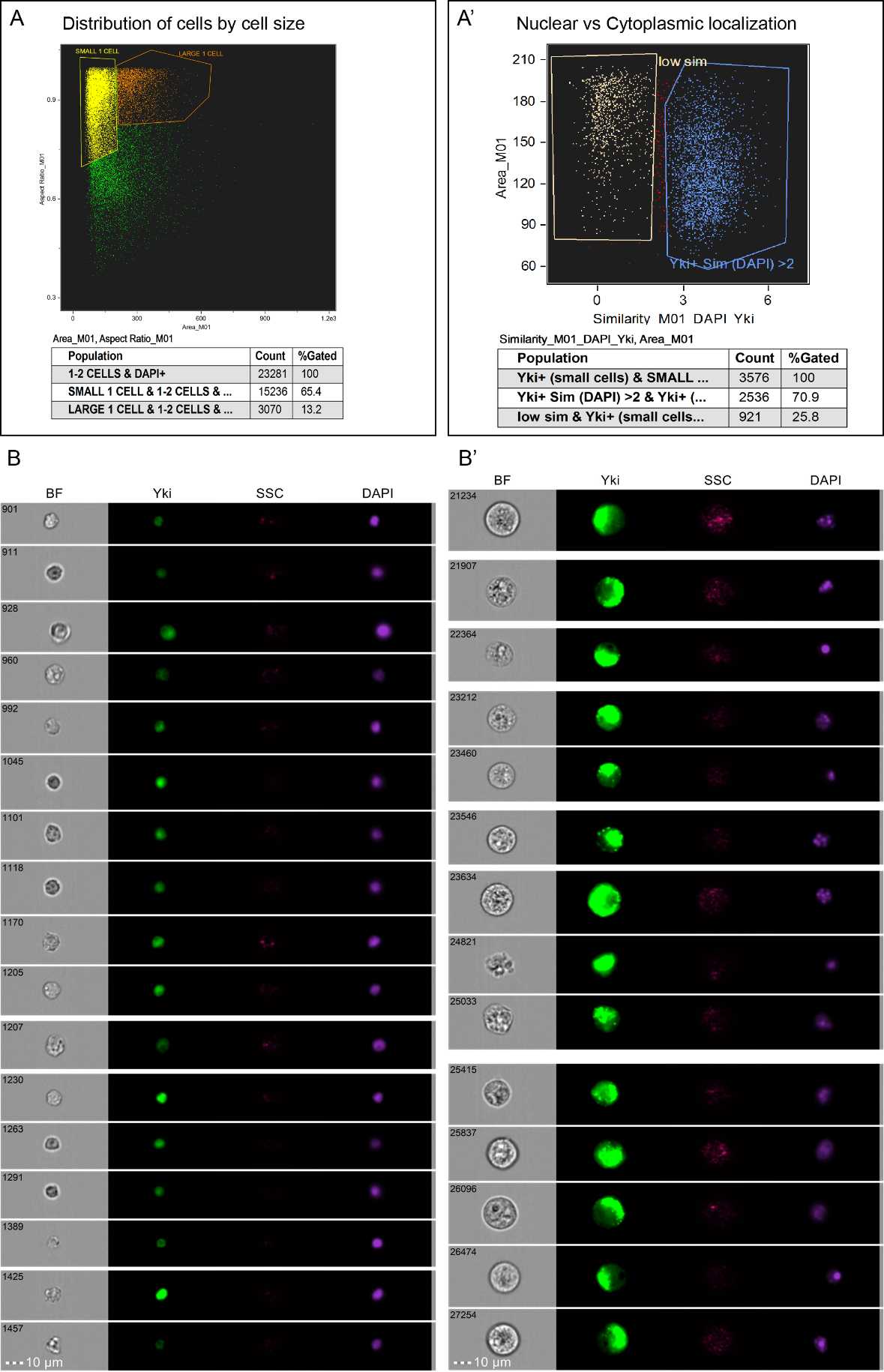


**Supplementary Figure 8.** **Flow Cytometric study of YAP expressing cells in *Hydra* using ImageStream.** The dissociated cells from *Hydra* were fixed and stained using anti-CheYki antibody and DAPI, for the DNA and analyzed by ImageStream. **(A)** Cell size distribution of *Hydra* cells were assessed for cell size-Large and Small based on the area feature. 200 µm^2^ area was used as a cut-off for small cells. **(A’)** The small cells were further gated for nuclear co-localization using the similarity feature. A similarity value greater than 2 of YAP expressing cells with DAPI stained nucleus was considered as co-localized. **(B)** Individual YAP nuclearized “Small” cells imaged by ImageStream displaying Brightfield (BF), YAP staining (Yki), Side scatter and DAPI staining in each column. **(B’)** Individual cytoplasmic YAP localized “Large” cells imaged by ImageStream displaying Brightfield (BF), YAP staining (Yki), Side scatter and DAPI staining in each column.


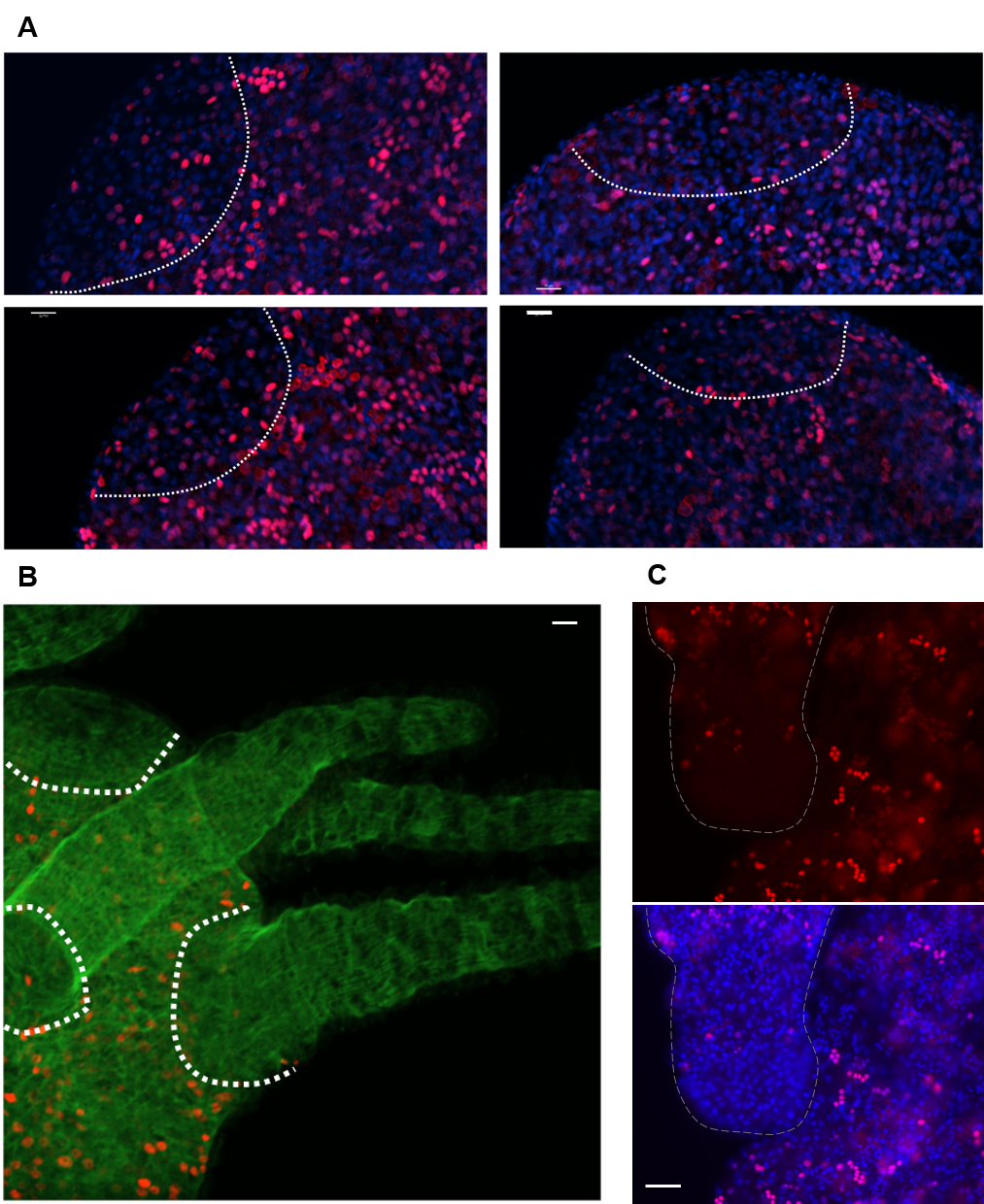


**Supplementary Figure 9.** **Immunofluorescence localization of *Hvul*_YAP.** The assay was performed using anti-CheYki antibody showing **(A)** YAP expressing cells at the distal tip of bud (future hypostomal region indicated by white dotted lines) non-clustered as compared to the rest of the lower bud region. Red: YAP & Blue: DAPI. (Scale bar = 50 µm) **(B)** At stage 9 onwards of budding, non-clustered YAP expressing cells were observed sparsely at the boundaries between the hypostome and the tentacle base**.** Red: YAP & Green: Actin. (Scale bar = 50 µm) **(C)** A lack of YAP expressing cells can be seen at the Adult-bud boundary (marked by dashed white line) where the future basal disk will form. The top image shows YAP expression and the bottom image shows merged image of YAP expression and nuclear stain. Red: YAP & Blue: DAPI (Scale bar = 50 µm).


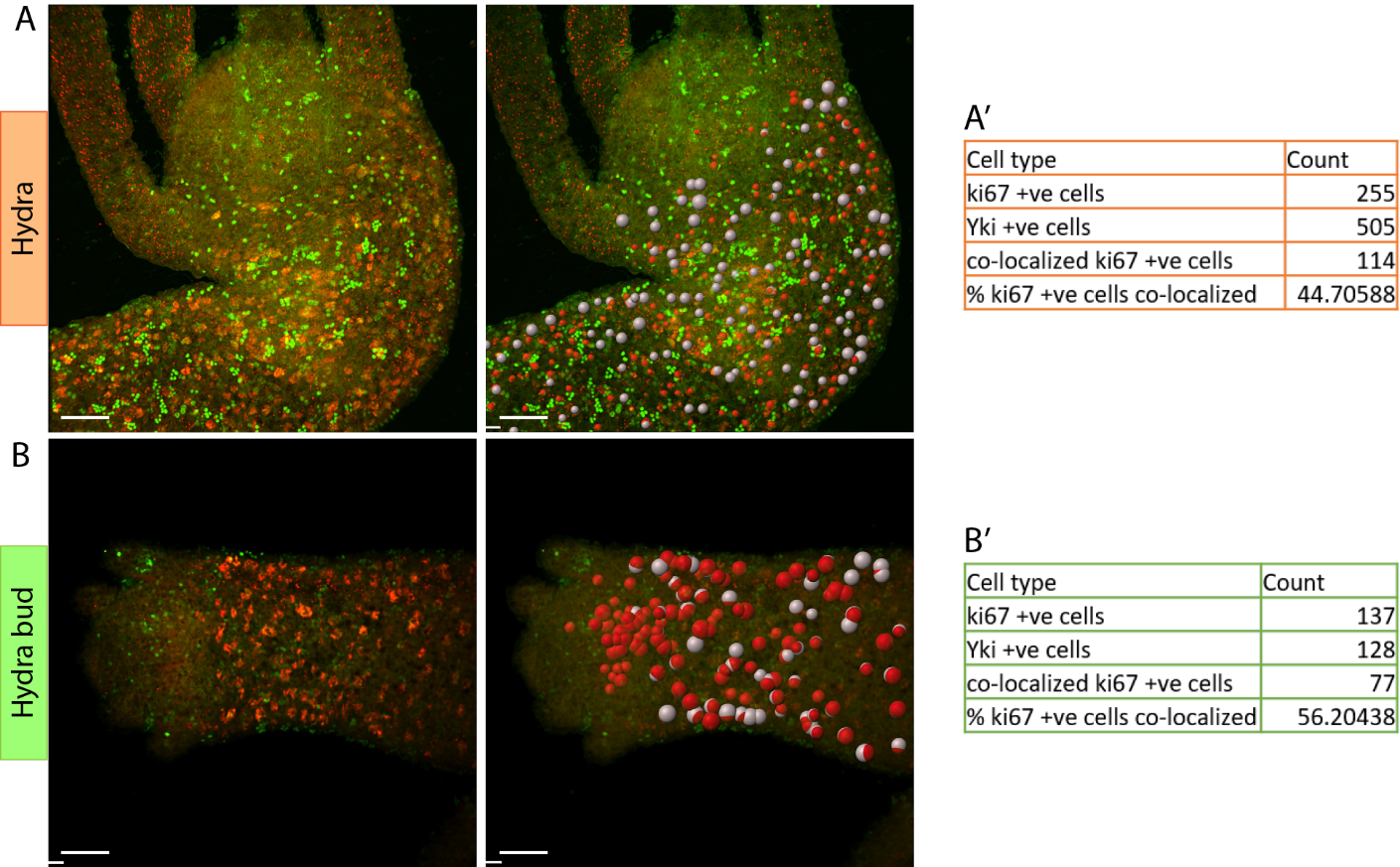


**Supplementary Figure 10.** **Colocalization analysis of cells expressing Ki67 and YAP indicates that almost half the proliferating cells in Hydra co-express YAP. A.** LEFT: A merged immunofluorescence image of an adult polyp expressing YAP (Green) and Ki67 (Red). RIGHT: Grey spherical spots are representing YAP expressing cells with dimensions similar to Ki67 expressing cells (red) and colocalized with Ki67 expressing cells. **A’.** Statistical data from the image analysis of Figure A showing counts of YAP expressing cells, Ki67 expressing cells and cells with colocalized expression of Ki67 and YAP. **B.** LEFT: A merged immunofluorescence image of a polyp bud expressing YAP (Green) and Ki67 (Red). RIGHT: Grey spherical spots are representing YAP expressing cells with dimensions similar to Ki67 expressing cells (red) and colocalized with Ki67 expressing cells. **B’.** Statistical data from the image analysis of Figure A showing counts of YAP expressing cells, Ki67 expressing cells and cells with colocalized expression of Ki67 and YAP.

Supplementary Method

**Colocalization analysis**

Colocalization analysis was performed using Imaris (ver 9.4). For the analysis, the spot feature was used to identify both Ki67 positive cells and YAP positive cells. Since there were too many cells that were YAP positive and we could visually infer that the co-localized cells had YAP staining being similar in size to that of Ki67 staining. The diameter of the Ki67 staining region was sampled randomly and was found to be an average of 10 µm (XY) and 6 µm (Z). Based on this information, spots were set with these parameters as a cut-off for generating spots for both YAP and Ki67 expressing cells. This also allowed weeding out tiny speckles in the image, which were either noise or non-specific signals. Once the spots were generated, colocalized signals were subset by adding the filter on Ki67 positive cells with minimal distance from the YAP positive cells. This distance was calculated by measuring the distance of nearby spheres (spot) from their geometrical center. The minimum distance cut-off was set as 4 µm. This filter was enough to encompass most of the co-localized cells needed for the analysis. Once this was set, Imaris provided the count of the cells in which co-localization was observed.
